# Advances in biomarkers for diagnosing and prognosticating disorders of consciousness

**DOI:** 10.3389/fnins.2026.1823376

**Published:** 2026-07-16

**Authors:** Xinyi Yang, Yunqian Luo, Zhiqun Chen, Yiyi Li, Li Pan, Pengfei Wang, Zhen Chen

**Affiliations:** 1 School of Rehabilitation Science, Shanghai University of Traditional Chinese Medicine, Shanghai, China; 2 Department of Rehabilitation Medicine, School of Medicine, Renji Hospital, Shanghai Jiao Tong University, Shanghai, China; 3 The Neurorehabilitation Centre, The First Rehabilitation Hospital of Shanghai, Shanghai, China; 4 Department of Rehabilitation Medicine, Shanghai Punan Hospital, Shanghai, China; 5 Shanghai Key Laboratory for Nucleic Acid Chemistry and Nanomedicine, School of Medicine, Renji Hospital, Institute of Molecular Medicine, Shanghai Jiao Tong University, Shanghai, China

**Keywords:** biomarkers, disorders of consciousness, gut-brain axis, metabolomics, miRNAs, proteomics, traumatic brain injury

## Abstract

Disorders of Consciousness (DoC) resulting from brain injury comprise a spectrum of clinical syndromes, where the level of impairment varies considerably depending on lesion location, etiology, severity, and individual patient factors. These differences substantially influence both rehabilitation strategies and long-term prognosis. Current diagnostic assessment relies primarily on behavioral scales, supplemented by electrophysiological and neuroimaging studies; however, these approaches remain limited in objectivity, sensitivity, and accessibility. There is therefore a clinical need for biologically informative biomarkers to improve diagnostic precision and prognostic stratification in DoC. This narrative review synthesizes recent advances in biomarker research, encompassing proteomic, metabolomic, and microRNAs (miRNAs) signatures, across multiple biological specimens. We evaluate the findings spanning exploratory to validation stages, and discuss their translational potential, providing a valuable reference for future large-scale, multicenter investigations.

## 1 Introduction

Consciousness arises from the integrated activity of brainstem and subcortical arousal systems with thalamocortical and cortico-cortical cognitive networks (Blumenfeld, 2023; Whyte et al., 2024). This integration enables the perception, synthesis and interpretation of both internal states and the external environment, expressed through volitional behavior, language and goal-directed actions. Disorders of consciousness (DoC) are characterized by a severe impairment in environmental awareness and responsiveness, which clinically manifests as a profound disruption in the capacity for sustained, selective, and integrative awareness, coupled with markedly diminished or inconsistent responsiveness (Giacino et al., 2014; Cambareri et al., 2025; Zhang et al., 2025a). DoC typically result from acquired brain injuries, with common etiologies including traumatic brain injury (TBI), hypoxic–ischemic brain injury (HIBI), and cerebrovascular events. TBI constitutes a leading cause of DoC (Kowalski et al., 2021; Johnson-Black et al., 2025). Severe trauma induces irreversible structural damage to brain tissue, impairing both the cerebral cortex and the ascending reticular activating system within the brainstem. This disrupts the anatomical integrity and functional connectivity of the neural circuits subserving consciousness, ultimately leading to a sustained depression of conscious level (Steyerberg et al., 2019; Li et al., 2024; Meng et al., 2023). The long-term care needs of patients with chronic DoC impose a substantial socioeconomic burden on families and healthcare systems, establishing DoC as a significant public health challenge (Zhang et al., 2021; Saporito et al., 2024; Corallo et al., 2021). The primary diagnostic categories within DoC are the Vegetative State (VS), also termed Unresponsive Wakefulness Syndrome (UWS), and the Minimally Conscious State (MCS). With advancing research, MCS has been subcategorized into MCS+ and MCS−, differentiated by the presence or absence of discernible language-related behaviors, respectively (Chen et al., 2025a; Thibaut et al., 2020). MCS+ is characterized by behaviors such as command-following, intelligible verbalization, or intentional communication, making this subtyping an important clinical distinction for diagnostic stratification and long-term prognostic evaluation. When patients recover consistent functional communication or object use, they are classified as having Emerged from the Minimally Conscious State (EMCS) (Edlow et al., 2021; Laureys et al., 2010; Golden et al., 2024; Zhang et al., 2025b). Furthermore, research has identified a subpopulation of patients demonstrating Cognitive Motor Dissociation (CMD), a novel dissociation characterized by reproducible neural responses to commands in the absence of overt behavioral output (Bodien et al., 2024). Critically, prognosis diverges significantly between these diagnostic entities, with outcomes for MCS being markedly more favourable than for VS/UWS (Lammi et al., 2005; Strauss et al., 2000; Luauté et al., 2010). Consequently, the accurate and early differential diagnosis of the specific level of consciousness impairment is of paramount importance. It informs prognostic stratification, guides the selection of targeted therapeutic and rehabilitative interventions, and shapes clinical management pathways.

In the clinical diagnosis of patients with DoC, the Coma Recovery Scale-Revised (CRS-R) remains the sole clinically recommended diagnostic gold standard (Giacino et al., 2004; Murtaugh and Shapiro Rosenbaum, 2023; Bodien et al., 2016). While it provides an initial assessment of a patient’s level of consciousness, the scale’s inherent subjectivity poses significant limitations. A single CRS-R assessment often fails to capture the fluctuating nature of a patient’s conscious state, and its results are susceptible to variability based on the examiner’s experience and spontaneous fluctuations in the patient’s arousal and attention (Wannez et al., 2017; Bodien et al., 2023). Consequently, objective measures such as electroencephalography (EEG) and functional magnetic resonance imaging (fMRI) are routinely employed as supplementary tools to corroborate the findings of subjective behavioral scales (Estraneo et al., 2020; Rossi Sebastiano et al., 2021; Gosseries et al., 2011). However, these neuroimaging and electrophysiological techniques also present constraints, including operational complexity, lengthy acquisition times, substantial costs, and contraindications for patients with metallic implants (Kazazian et al., 2025). Recently, the development of biomarkers derived from patient biofluids and other biospecimens has emerged as a prominent research frontier in neurological disorders (Ye et al., 2021; Muñoz-San Martín et al., 2023; Nilsson et al., 2024, Martinez and Peplow, 2025). Blood-based biomarkers have gained increasing clinical relevance in brain injury assessment. The American College of Surgeons (2024) Best Practices Guidelines for Traumatic Brain Injury discuss blood-based biomarkers, including Glial Fibrillary Acidic Protein (GFAP), Ubiquitin C-Terminal Hydrolase-L1 (UCH-L1), and S100 Calcium Binding Protein Beta (S100B), as adjunctive tools for imaging decisions and outcome prediction after TBI. Moreover, FDA-cleared blood-based tests measuring GFAP and UCH-L1 are available for the evaluation of adults with suspected mild TBI, primarily to help rule out computed tomography (CT)-detectable acute intracranial lesions and reduce unnecessary CT imaging (Bazarian et al., 2021; U.S. Food and Drug Administration, 2018). Recent recommendations further support the incorporation of blood-based biomarkers into acute TBI characterization, risk stratification, prognostic assessment, and research classification frameworks (Bazarian et al., 2025). These advances provide an important translational foundation for exploring blood-based biomarkers in severe brain injury and DoC. Nevertheless, because most guideline-supported applications are derived from acute TBI cohorts, particularly mild-to-moderate TBI populations, their diagnostic and prognostic value in Prolonged Disorders of Consciousness (pDoC) and non-traumatic etiologies requires further prospective validation. More broadly, biofluid-based biomarkers may provide objective, accessible, and mechanistically informative data to complement behavioral, neuroimaging, and electrophysiological assessments in DoC. Despite this progress, comprehensive reviews that integrate findings across multiple biological sample types specifically for DoC are scarce. Previous reviews have often focused on single biomarker categories, restricted biospecimen sources, or broader TBI-related biomarker evidence, including blood/cerebrospinal fluid (CSF) biomarkers, salivary miRNAs, and injury-related molecular markers (Wang et al., 2016; Sessa et al., 2025; Luo et al., 2025; Friberg et al., 2024; Bagnato and Boccagni, 2023; Migdady et al., 2025; Hiskens et al., 2022). Therefore, an integrated synthesis across central, peripheral, and intestinal biospecimen sources may help clarify the current evidence landscape and translational gaps in DoC biomarker research.

**Figure 1:**
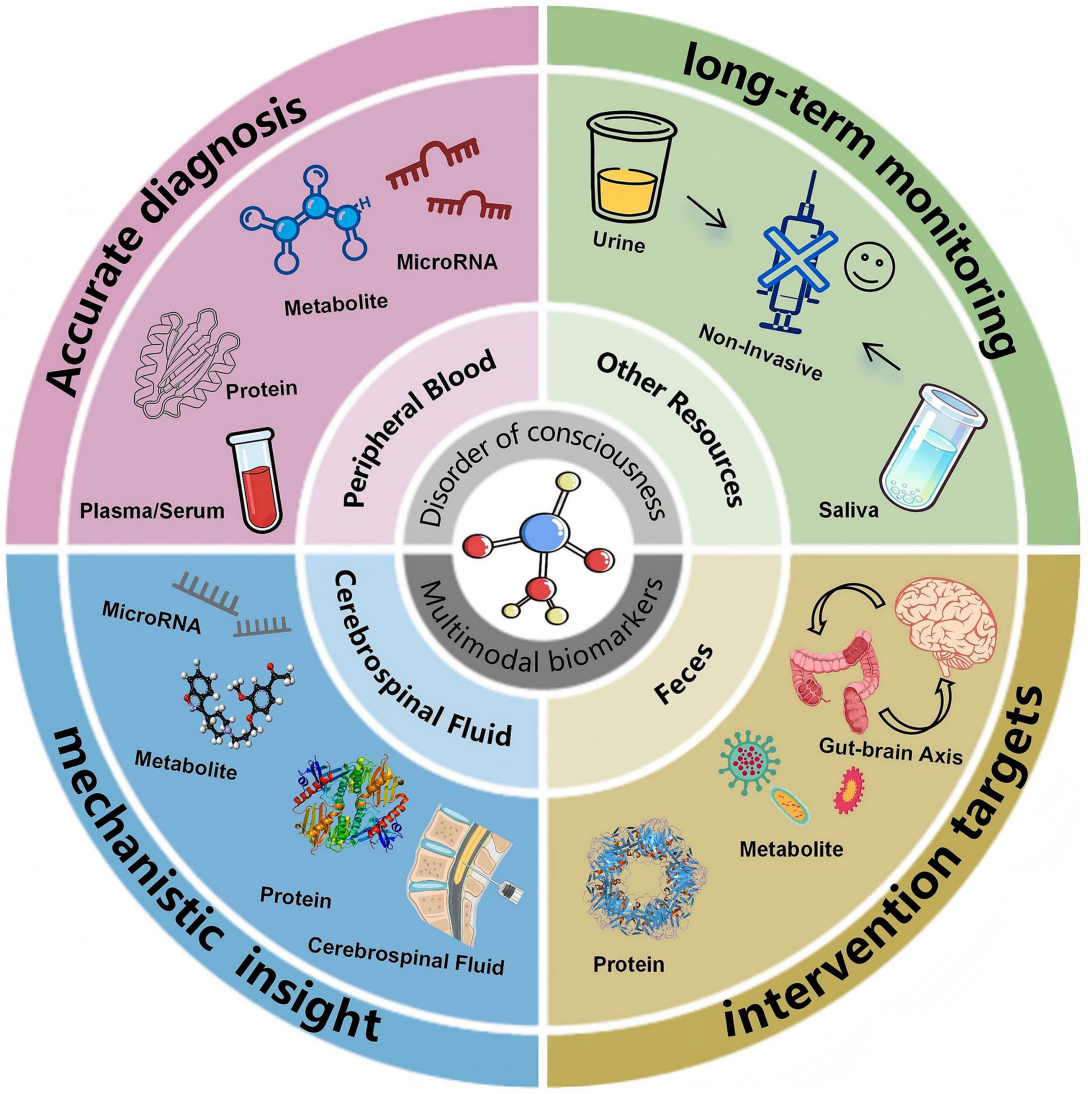
Schematic overview of advances in biomarkers for diagnosing and prognosticating disorders of consciousness. This schematic summarizes four complementary biomarker research domains in disorders of consciousness (DoC): peripheral blood for accessible diagnosis; cerebrospinal fluid for mechanistic insight; feces for intervention targets; and other non-invasive resources, including urine and saliva, for long-term monitoring. Key biomarker classes include proteins, metabolites, miRNAs.

This review aims to summarize current biomarker evidence in DoC across multiple biospecimen sources, including peripheral blood, CSF, feces/stool, urine, and saliva. To move beyond a simple catalogue of individual biomarkers, we adopt a DoC-oriented “central–peripheral–intestinal” framework that connects central nervous system injury, peripheral inflammatory and metabolic responses, and gut-brain-axis-related alterations with key clinical challenges in diagnosis, prognosis, and translation. We focus on major biomarker classes, including proteins, metabolites, and microRNAs (miRNAs), and discuss their diagnostic, prognostic, mechanistic, and translational relevance. Accordingly, the review is organized around four complementary domains: peripheral blood for accessible biomarker assessment, CSF for central nervous system-related information, the gut-brain axis (GBA) for intestinal and systemic interactions, and non-invasive samples for feasible longitudinal monitoring. Within each domain, representative biomarkers and current evidence limitations are discussed to provide a reference for future biomarker validation and translational research in DoC (Figure 1).

### 1.1 Literature search and selection strategy

This article was designed as a narrative review to summarize evidence on biofluid- and biospecimen-based biomarkers for the diagnosis and prognosis of DoC. To support the narrative synthesis, a targeted literature search was conducted in PubMed, Web of Science, CNKI, and Wanfang Data from database inception to the time of the revised manuscript preparation. The search strategy combined subject headings, where applicable, and free-text keywords related to DoC, brain injury, biological sample types, and biomarker categories. The main search terms included “disorders of consciousness,” “DoC,” “prolonged disorders of consciousness,” “pDoC,” “vegetative state,” “unresponsive wakefulness syndrome,” “minimally conscious state,” “MCS,” “traumatic brain injury,” “hypoxic–ischemic brain injury,” “biomarker,” “serum,” “plasma,” “blood,” “cerebrospinal fluid,” “feces,” “stool,” “urine,” “saliva,” “proteomics,” “metabolomics,” “microRNA,” “miRNA,” “gut microbiota,” and “gut-brain axis.” Detailed search terms and representative database-specific search combinations are provided in Supplementary Table S1.

The database search identified 10,600 records, including 6,416 from Web of Science, 2,843 from PubMed, 852 from Wanfang Data, and 489 from CNKI. After 5,982 duplicate records were removed manually, 4,618 records were screened by title and abstract. Of these, 4,469 records were excluded because they were unrelated to the review topic, did not report biological biomarker data, or represented overlapping datasets. A total of 149 reports were sought for retrieval, of which 74 could not be retrieved. These non-retrieved reports mainly consisted of records for which full texts were unavailable through institutional access or public databases, conference abstracts without accessible full-text articles, or publications with insufficient bibliographic information for retrieval. Because these reports could not be assessed in detail, they were not included in the core narrative synthesis, which may have introduced a degree of selection bias. Therefore, the conclusions of this narrative review should be interpreted in light of this limitation. The remaining 75 full-text reports were assessed for eligibility, and 45 were excluded because they focused only on imaging, electrophysiology, behavioral assessment, or rehabilitation interventions without biospecimen-based biomarker data; were abstracts, letters, or guidelines without original biomarker data; or lacked sufficient study data for interpretation. Finally, 30 reports were included in the narrative synthesis.

Study selection was conducted by two authors. Titles and abstracts were first screened independently by two authors according to the predefined article consideration and exclusion criteria. Potentially relevant reports were then assessed at the full-text level by the same two authors. Disagreements during title/abstract screening or full-text assessment were resolved through discussion, and when consensus could not be reached, a third author was consulted.

Articles were considered if they: (1) involved patients with DoC, prolonged DoC, VS/UWS, MCS, EMCS, or brain-injury populations relevant to DoC biomarker research; (2) investigated biomarkers derived from blood, cerebrospinal fluid, feces/stool, urine, saliva, or other biological specimens; (3) reported diagnostic, prognostic, etiological, pathophysiological, or translational relevance; or (4) provided methodological, mechanistic, guideline-based, or translational context for biomarker research in brain injury and DoC.

Articles were generally excluded if they: (1) were unrelated to DoC, brain injury, or biomarker research; (2) focused exclusively on neuroimaging, electrophysiology, behavioral assessment, or rehabilitation interventions without biological biomarker data; (3) did not involve blood, cerebrospinal fluid, feces/stool, urine, saliva, or other biospecimen-based biomarkers; (4) lacked sufficient methodological details, population information, or outcome data to support interpretation; (5) represented duplicate publications or overlapping datasets, in which case the most complete or most relevant report was retained; or (6) were conference abstracts without full text, editorials, letters, news articles, or commentaries without original data. Reviews, guidelines, consensus statements, and methodological articles were used only when they provided background information, clinical guidance, or methodological context, and were not considered core biomarker evidence.

**Figure 2:**
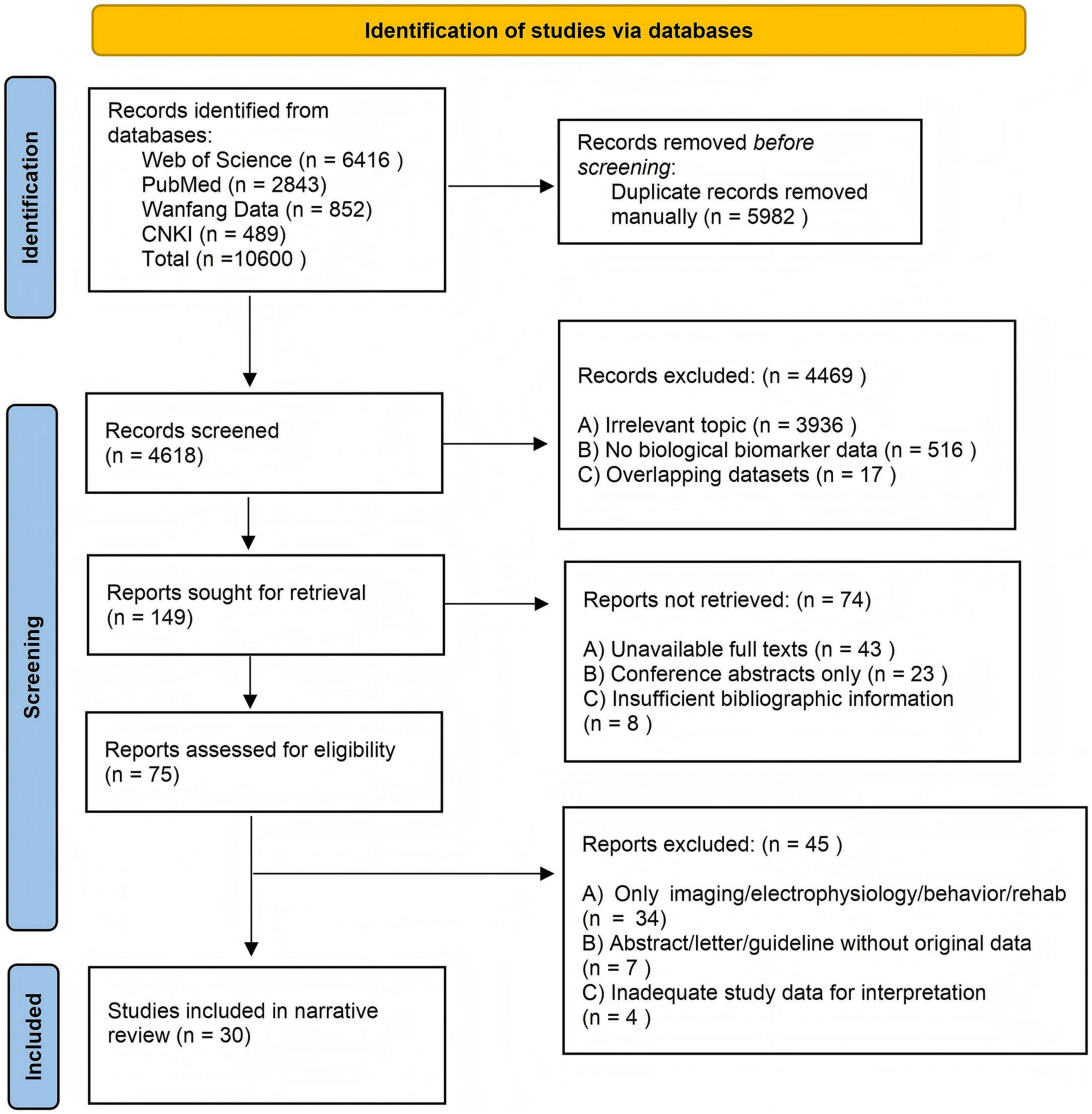
Literature screening flow diagram. The flow diagram summarizes the identification, screening, eligibility assessment, and inclusion process of studies for this narrative review.

Original studies were prioritized when discussing diagnostic or prognostic biomarker performance. The articles discussed in the main text, figures, and summary table were selected based on relevance to the topic, study design, directness of evidence for DoC, biomarker type, validation status, and contribution to the multi-compartment biomarker framework of this review. The literature identification and selection process is summarized in Figure 2, while the conceptual framework of the narrative synthesis is provided in Supplementary Figure S2.

## 2 Peripheral blood-based biomarkers

**Figure 3:**
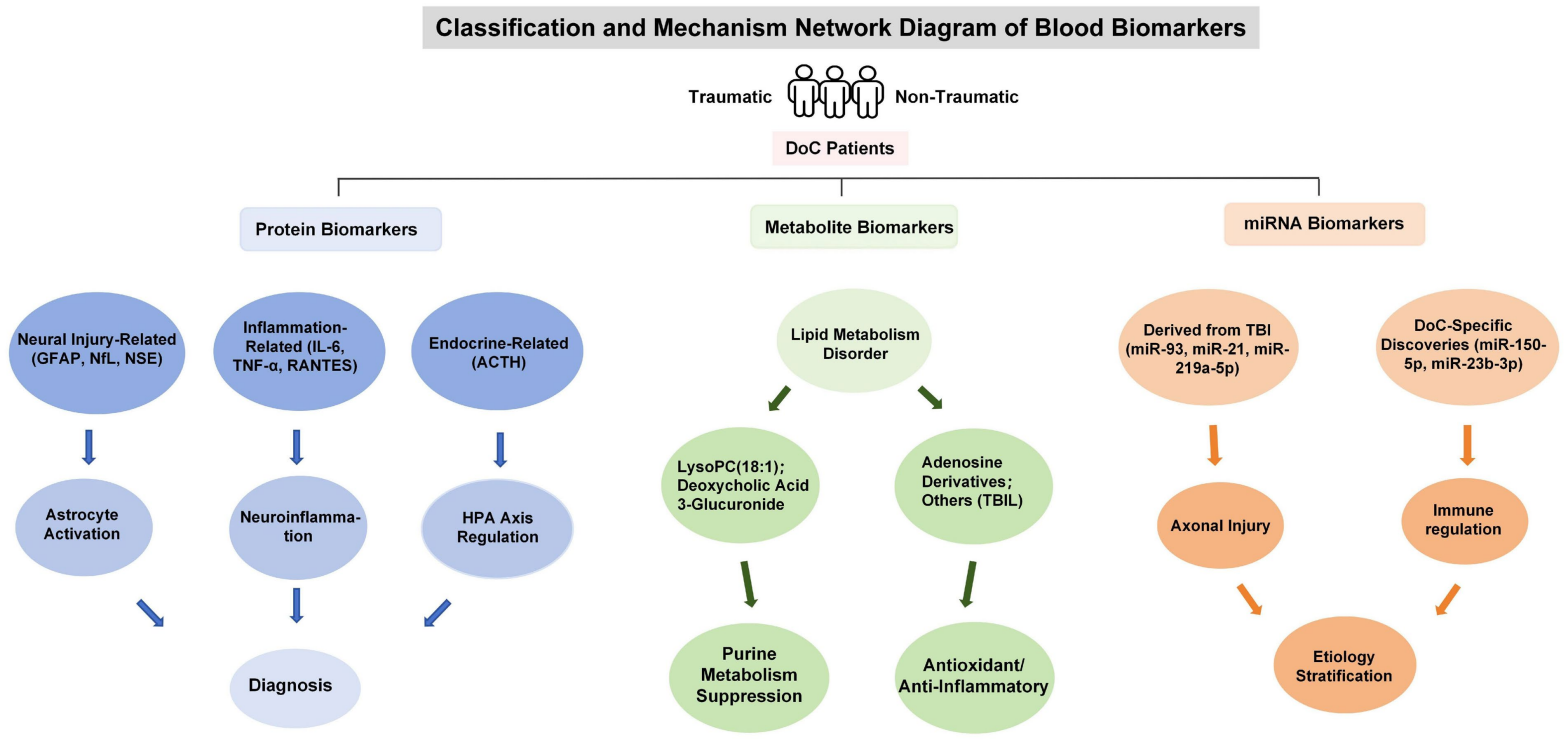
Classification and proposed mechanistic network of peripheral blood biomarkers in disorders of consciousness. This schematic summarizes the classification and proposed mechanism network of blood biomarkers in traumatic and non-traumatic disorders of consciousness (DoC) patients. Blood biomarkers are grouped into protein biomarkers, metabolite biomarkers, and miRNA biomarkers. Protein biomarkers include neural injury-related markers (GFAP, NfL, NSE), inflammation-related markers (IL-6, TNF-α, RANTES), and endocrine-related markers (ACTH), which are linked to astrocyte activation, neuroinflammation, HPA axis regulation, and diagnosis. Metabolite biomarkers are presented in relation to lipid metabolism disorder, including LysoPC(18:1), deoxycholic acid 3-glucuronide, adenosine derivatives, and TBIL, which are linked to purine metabolism suppression and antioxidant/anti-inflammatory pathways. miRNA biomarkers are divided into TBI-derived candidates (miR-93, miR-21, miR-219a-5p) and DoC-specific discoveries (miR-150-5p, miR-23b-3p), which are linked to axonal injury, immune regulation, and etiology stratification.

As the most readily accessible, minimally invasive, and serially obtainable biological specimen, peripheral blood (specifically plasma or serum), occupies a pivotal role in biomarker research for DoC. Molecular constituents in peripheral blood including proteins, metabolites, and circulating miRNAs, can serve as reflective indicators of central nervous system (CNS) pathophysiology. Given that TBI represents a primary etiology of DoC, the robust foundation of clinical evidence established in TBI blood biomarker studies provides substantial support for cross-etiological investigations into DoC biomarkers. Because many blood-based biomarkers were initially identified or validated in acute TBI cohorts, their relevance to pDoC should be interpreted according to etiology, disease stage, sampling time, and validation context. Direct extrapolation to hypoxic–ischemic, stroke-related, or mixed-etiology DoC remains inappropriate without dedicated cohort validation. This analysis is structured around three core molecular domains: proteomics, metabolomics, and miRNA profiling, as visually summarized in Figure 3. Across these domains, the evidence base ranges from relatively better-characterized protein assays to more exploratory metabolomic and miRNA signatures that require further validation. Overall, these biomarkers—by reflecting or being associated with key pathological processes such as neuroinflammation, axonal injury, metabolic dysregulation, and hypothalamic–pituitary–adrenal (HPA) axis dysfunction—contribute to diagnostic stratification, prognosis prediction, and etiological differentiation.

### 2.1 Protein biomarkers in peripheral blood

Peripheral blood protein biomarkers provide one of the most clinically accessible approaches for molecular assessment in DoC. However, their translational relevance requires careful interpretation, because many circulating proteins initially studied in TBI primarily reflect general brain injury burden rather than impaired consciousness itself. These markers are biologically relevant to DoC, but their specificity depends on whether they capture tissue-level injury, systemic responses, or mechanisms more directly related to consciousness-related network dysfunction.

From a mechanistic perspective, peripheral blood protein biomarkers can be grouped into three major categories. First, structural neural injury markers, including Neurofilament Light Chain (NfL), tau protein, UCH-L1, and Neuron-Specific Enolase (NSE), mainly reflect axonal or neuronal damage (Agoston et al., 2017; Yokobori et al., 2013). Second, glial and blood–brain barrier-related markers, such as GFAP and S100B, indicate astroglial activation, glial injury, or barrier disruption (Agoston et al., 2017; Yokobori et al., 2013). Third, inflammatory and endocrine-related markers, including interleukin-6 (IL-6), tumor necrosis factor-α (TNF-α), interleukin-13 (IL-13), RANTES, and adrenocorticotropic hormone (ACTH), may reflect secondary inflammatory activation, systemic stress responses, or recovery-related neuroendocrine regulation. This mechanistic classification is important because these processes may influence consciousness-related biology through disruption of long-range neural connectivity, secondary neuroinflammation, blood–brain barrier dysfunction, altered synaptic environments, and systemic stress responses. However, they do not necessarily distinguish impaired consciousness from the broader consequences of brain injury.

Among currently available candidates, NfL appears to have the most direct relevance to pDoC stratification. Serum NfL has been reported to remain elevated in patients with pDoC at 1–3 and 6 months after injury, with associations with consciousness level, including higher levels in UWS than in MCS during the early phase, and with etiology, including higher levels in HIBI than in TBI during later stages (Bagnato et al., 2021). These findings suggest that NfL may reflect persistent axonal degeneration and disease-stage-related injury burden in pDoC. However, whether NfL is specific to impaired consciousness rather than persistent axonal injury remains to be determined.

Other neuronal and glial injury markers provide supportive but less DoC-specific information. NSE has been found to be significantly elevated in individuals with repetitive head trauma even after a 2-month rest period, as detected by biochip array technology, suggesting persistent neuronal damage independent of glial pathology (Zetterberg et al., 2009). GFAP and UCH-L1 have stronger translational foundations in TBI research than in DoC-specific validation. GFAP, an astrocyte-specific glial biomarker, has shown higher diagnostic specificity than S100B for differentiating traumatic intracranial lesions in quantitative serum enzyme-linked immunosorbent assay (ELISA) studies (Papa et al., 2014). It may also help characterize astroglial injury, blood–brain barrier disruption, and white matter microstructural damage, as serum GFAP levels measured using ultrasensitive single-molecule array (Simoa) technology have been associated with white matter hyperintensity volume and fractional anisotropy on neuroimaging (Coppola et al., 2022). In contrast, S100B is upregulated in the early phase of TBI but may be confounded by extracranial injury or hepatic dysfunction, limiting its disease specificity (Al-Adli et al., 2021). UCH-L1 may provide complementary information on neuronal injury; ELISA-based studies have detected elevated UCH-L1 levels in the serum and CSF of severe TBI patients, with levels associated with injury severity and mortality (Mondello et al., 2012). Nevertheless, these markers should currently be interpreted primarily as brain-injury-related rather than DoC-specific biomarkers. Their future value may lie in combination with NfL, inflammatory mediators, and clinical or neurophysiological variables rather than in standalone diagnosis.

Inflammatory-related proteins also hold prognostic value, but their biological non-specificity limits interpretation. IL-6, TNF-α, and RANTES in peripheral blood are significantly upregulated following severe TBI. Animal models have delineated the spatiotemporal dynamics of TNF-α and IL-6, while prospective human studies have shown that plasma RANTES correlates with early mortality (Yu, 2008; Albert et al., 2017). In pDoC cohorts, IL-13 and TNF-α have shown associations with CRS-R scores and long-term functional recovery, suggesting potential relevance for prognostic stratification (Wu et al., 2021). However, cytokines and chemokines are strongly influenced by infection, systemic inflammation, medication exposure, nutritional status, and comorbidities. Similarly, ACTH may reflect hypothalamic–pituitary–adrenal axis activity, but its direct relationship with consciousness recovery remains uncertain (Wu et al., 2024).

Despite these advances, critical gaps persist in the field. Most studies are small-scale and exhibit substantial methodological heterogeneity. First, differences in assay sensitivity may lead to inconsistent reports of biomarker concentrations across studies. Conventional ELISA-based assays generally have lower analytical sensitivity than ultrasensitive Simoa platforms, which can detect low-abundance proteins such as NfL at the pg./mL level. Second, non-standardized sampling timelines, with blood collected at varying intervals after injury, may confound the associations between protein levels and clinical outcomes. Third, protein expression can be influenced by confounding factors such as extracranial injury or hepatic disease, further complicating cross-study comparisons. Overall, peripheral blood protein biomarkers should not be presented as a homogeneous group with equivalent translational maturity. Current evidence suggests that NfL is among the most directly supported circulating markers for pDoC-related stratification, whereas GFAP and UCH-L1 are better positioned as brain-injury-related markers with potential but still unproven DoC-specific value. Inflammatory cytokines may complement prognostic assessment, while NSE, S100B, RANTES, and ACTH remain supportive or context-dependent candidates. Future studies should include brain-injured control groups without persistent DoC, stratify patients by etiology and consciousness phenotype, and evaluate the incremental diagnostic or prognostic value of protein panels beyond established behavioral, electrophysiological, neuroimaging, and clinical prognostic assessments.

### 2.2 Metabolite biomarkers in peripheral blood

Beyond protein biomarkers that mainly reflect neural injury and inflammatory responses, blood metabolomics offers a comprehensive view of systemic metabolic perturbations in DoC patients. By profiling small-molecule metabolites, this approach can capture changes in lipid, amino acid, and purine metabolism that are not fully revealed by protein markers, thereby providing complementary information for etiological stratification, consciousness-level differentiation, and prognostic prediction in DoC (Orešič et al., 2016).

**Figure 4:**
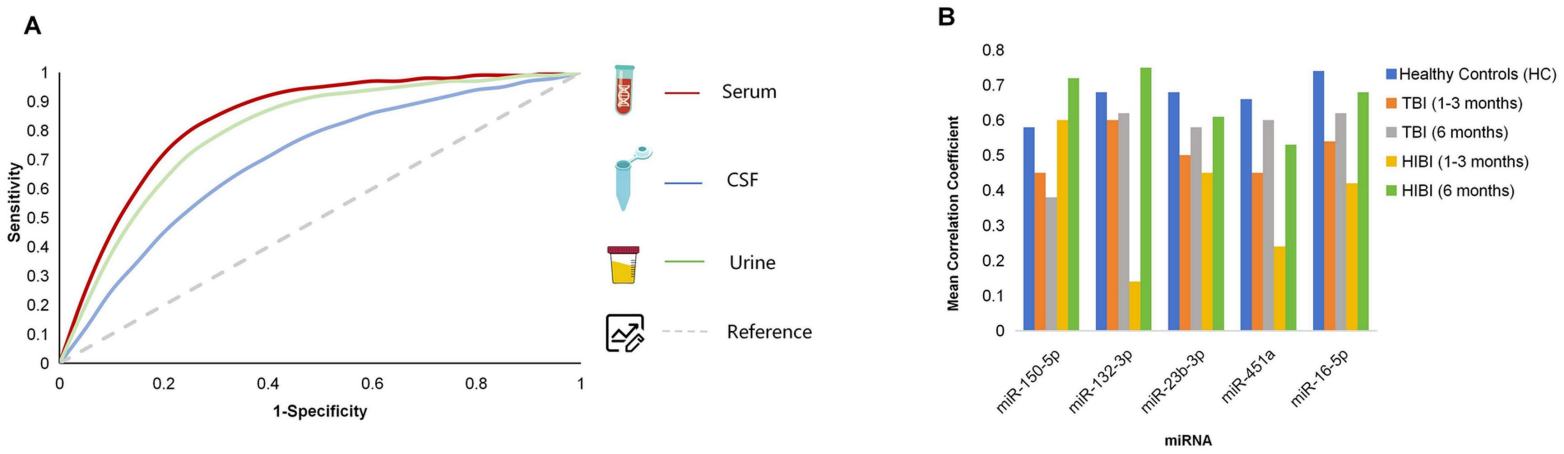
The detection of metabolite biomarkers and microRNA biomarkers in peripheral blood. **(A)** Diagnostic performance of metabolite panels derived from different biofluids for distinguishing UWS from MCS (Wang et al., 2025). Copyright: 2025, John Wiley & Sons, Inc. **(B)** Mean Correlation Coefficients of Circulating miRNA Expression in TBI/HIBI Patients vs. Healthy Controls (Musso et al., 2023). Copyright: 2023, Springer Nature.

Current serum metabolomic studies suggest that metabolic dysregulation in DoC is not limited to a single pathway, but instead involves several recurring biological themes. Studies using ultra-high-performance liquid chromatography coupled with quadrupole-Orbitrap high-resolution mass spectrometry (UHPLC-Q-Orbitrap HRMS) have reported alterations in amino acid and lipid metabolism in TBI-induced DoC, including 53 differential metabolites across 10 core metabolic pathways (Feng et al., 2024). Similarly, an untargeted ultra-performance liquid chromatography–tandem mass spectrometry (UPLC–MS/MS) analysis distinguished patients with UWS from MCS based on 24 differential serum metabolites (Wang et al., 2025), with lysophospholipids such as LysoSM (18:1) and LysoPC (18:1) increased in MCS patients and deoxycholic acid 3-glucuronide decreased. A serum panel composed of DG (34:1), PE (18:1), and isoproturon achieved a high diagnostic area under the curve (AUC) of 0.94 for differentiating UWS from MCS (Figure 4A). These findings indicate that serum metabolites may capture biological differences related to consciousness level. However, because this performance was derived from internal validation within the same dataset, it should be interpreted as hypothesis-generating rather than evidence of clinical readiness.

Targeted metabolomics provides a complementary strategy by focusing on predefined pathways with higher analytical specificity. Yu et al. reported abnormalities in 21 metabolic pathways in DoC, with purine metabolism being the most significantly suppressed. Reduced adenosine derivatives and elevated phosphatidylcholine (38:5)-H and arachidonic acid helped distinguish UWS from MCS and correlated positively with CRS-R scores (Yu et al., 2021). These findings suggest that predefined metabolic pathways may provide useful stratification signals for distinguishing consciousness levels in DoC. However, whether these metabolic changes specifically reflect impaired consciousness rather than broader consequences of brain injury, systemic illness, or prolonged immobilization remains uncertain and requires validation in appropriately controlled cohorts.

Mechanistically, the reported serum metabolic changes may reflect several biological processes relevant to DoC. Lipid and glycerophospholipid disturbances may indicate membrane remodeling, myelin or axonal injury, mitochondrial dysfunction, and inflammatory lipid signaling after severe brain injury (Adibhatla and Hatcher, 2008; Anthonymuthu et al., 2018; Sarkar and Lipinski, 2024). Suppressed purine metabolism may be related to impaired ATP turnover, altered adenosine signaling, and reduced energy-dependent synaptic activity, which are biologically relevant to sleep–wake regulation and arousal modulation (Basheer et al., 2004; Porkka-Heiskanen and Kalinchuk, 2011; Chikahisa and Séi, 2011). Amino acid metabolic changes may further reflect neurotransmitter imbalance, glutamate-related excitotoxic stress, or altered energy substrate utilization (Yi and Hazell, 2006; Lau and Tymianski, 2010). These pathways provide biologically plausible links between systemic metabolic dysregulation and impaired consciousness. However, their role in DoC pathophysiology remains uncertain, as they may either contribute to disease mechanisms or reflect secondary injury-related metabolic responses. Further validation through paired serum–CSF and longitudinal multimodal analyses is needed.

Recent studies have further examined whether specific serum metabolic indicators and profiles differ across etiological and prognostic subgroups. Huang et al. (2024) reported that total bilirubin (TBIL), a routine biochemical indicator with antioxidant and anti-inflammatory properties, was positively associated with prognosis in DoC patients, suggesting that clinically accessible metabolic markers may have auxiliary prognostic value. In addition, Ge et al. (2024) used liquid chromatography–mass spectrometry (LC–MS)-based untargeted metabolomics to characterize serum metabolic profiles across etiology, consciousness level, and prognosis. Their findings suggested lower levels of several glycerophospholipids in traumatic DoC than in non-traumatic DoC, distinct energy metabolic patterns across consciousness levels, and prognosis-related alterations such as reduced LysoPE (18:0/0:0) in patients with favorable outcomes. These findings suggest that serum metabolic profiles may help identify etiologically and prognostically relevant subgroups within DoC, but their DoC specificity and incremental value beyond established multimodal assessments remain to be established.

From a translational perspective, peripheral blood metabolomics remains largely in the discovery phase. Untargeted platforms differ in metabolite coverage, sensitivity, identification confidence, and quantitative precision. For example, Wang et al. (2025) used UPLC-MS/MS to identify serum metabolites distinguishing UWS from MCS, whereas Feng et al. (2024) adopted UHPLC-Q-Orbitrap HRMS to characterize lipid and amino acid metabolites in TBI-induced DoC, which may partly explain inconsistencies in the screening of core biomarkers. In addition, serum centrifugation conditions differ across studies, with some using 1,500 rpm for 15 min and others 3,000 rpm for 10 min, potentially contributing to variation in measured metabolite concentrations (Wang et al., 2025; Ge et al., 2024). Together with differences in sample handling, storage, freeze–thaw cycles, data-processing pipelines, normalization strategies, and statistical thresholds, these methodological variations remain a major barrier to developing a unified and reproducible serum metabolomic signature for DoC. Therefore, high-performing metabolite panels should currently be regarded as hypothesis-generating rather than clinically established. Their clinical value requires standardized workflows, external validation, and evidence of incremental value beyond established assessments.

### 2.3 MicroRNA biomarkers in peripheral blood

In addition to metabolic profiling that delineates downstream systemic changes, circulating miRNAs serve as key regulators of gene expression and may link upstream molecular pathways, such as apoptosis and neuroinflammation, to clinical phenotypes of DoC (Treiber et al., 2019). Owing to their relative stability in circulation and potential tissue specificity, miRNA biomarkers may complement metabolic and protein markers by reflecting etiological heterogeneity and temporal dynamics of brain injury, thereby enriching multimodal biomarker panels for DoC assessment (Wang et al., 2025). However, their translational value should not be overestimated. Circulating miRNA changes may arise from multiple cellular sources, including injured neural tissue, immune cells, endothelial cells, platelets, and other peripheral tissues. Therefore, an altered circulating miRNA signal should be interpreted as a systemic regulatory signature potentially related to brain injury and recovery, rather than as direct evidence of impaired consciousness.

Because direct miRNA evidence in DoC remains limited, methodological experience from TBI research provides an important starting point for DoC studies, while also highlighting several obstacles that directly affect clinical translation (Redell et al., 2010). Early studies established stem-loop quantitative reverse transcription-polymerase chain reaction (qRT-PCR) as a primary method for sensitive miRNA detection (Yang et al., 2016). Subsequent work introduced longitudinal sampling, TaqMan Low-Density Arrays (TLDA), digital polymerase chain reaction (dPCR), and in silico pathway analyses to improve temporal profiling, quantification accuracy, and biological interpretation (Schindler et al., 2020; Di Pietro et al., 2017; Yan et al., 2019; Sessa et al., 2025). Nevertheless, the lack of universally stable endogenous reference genes remains a major limitation across patient cohorts and biofluids (Chen et al., 2024). Different normalization strategies, including endogenous controls and exogenous spike-in controls, may substantially affect reported expression levels and limit cross-study comparability. Therefore, miRNA biomarkers should not be evaluated only by statistical significance or diagnostic performance; their reliability depends on standardized specimen type, preprocessing procedures, RNA extraction methods, normalization strategies, quantification platforms, and external validation.

#### 2.3.1 MiRNA biomarkers for traumatic brain injury research

TBI-related miRNA studies provide methodological references and candidate molecules for DoC research, but they should be interpreted as indirect evidence rather than DoC-specific validation. Previous studies have identified several circulating miRNAs associated with TBI diagnosis, injury severity, temporal evolution, or long-term outcome, including miR-93, miR-191, miR-499, miR-142-3p, miR-423-3p, miR-425-5p, miR-21, and miR-219a-5p (Yang et al., 2016; Schindler et al., 2020; Di Pietro et al., 2017; Yan et al., 2019). In silico analyses further suggest that dysregulated miRNAs may be involved in apoptosis, inflammation, cell survival, and PI3K/AKT-related pathways, providing plausible mechanistic hypotheses for subsequent DoC studies (Sessa et al., 2025).

The main value of these TBI-derived findings lies in technical and conceptual transfer rather than direct clinical applicability to DoC. They demonstrate how circulating miRNAs can be screened, quantified, longitudinally tracked, and linked to injury-related molecular pathways. However, most of these studies were conducted in acute TBI cohorts, where miRNA changes may primarily reflect acute tissue damage, blood–brain barrier disruption, systemic inflammation, or trauma-related stress responses. Such findings cannot be directly extrapolated to pDoC, hypoxic–ischemic injury, stroke-related DoC, or mixed etiologies. For DoC research, the key question is not whether a miRNA changes after brain injury, but whether it provides information about consciousness phenotype, recovery trajectory, or residual network function beyond injury severity and etiology. Thus, TBI-derived miRNAs should currently be regarded as candidate references for panel construction rather than validated DoC biomarkers.

#### 2.3.1 Emerging miRNA findings specific to DoC

Compared with TBI-derived evidence, DoC-focused miRNA studies provide more direct but still preliminary support for miRNA-based stratification. A longitudinal multicenter study showed that the expression patterns of miR-150-5p, miR-132-3p, miR-23b-3p, miR-451a, and miR-16-5p in the serum of DoC patients are related to the etiology and time after injury. In TBI-related DoC, baseline levels of miR-150-5p, miR-132-3p, and miR-23b-3p were significantly lower than those of controls, with high expression of the latter two linked to better outcomes. In HIBI-related DoC, miR-150-5p remained decreased at baseline and 6 months, and miR-451a was reduced at baseline, suggesting etiology-specific and time-dependent miRNA dynamics (Musso et al., 2023; Figure 4B). This study provides relatively stronger evidence because of its longitudinal multicenter design and dPCR-based quantification. However, it focused on a predefined limited panel, which improves validation strength but restricts discovery of novel DoC-related candidates and limits broader mechanistic inference.

Other DoC-related studies highlight both the potential and limitations of circulating miRNA profiling. Petrova et al. (2022) reported differential expression of miR-21, miR-93, miR-191, miR-let-7b, and miR-499 in plasma and CSF from pDoC patients, with miR-93, miR-21, and miR-191 showing pronounced differences across etiologies. This suggests that miRNA profiles may be more sensitive to etiological heterogeneity than to consciousness state alone. However, the absence of paired plasma–CSF correlation analysis limits interpretation of whether peripheral miRNA signals reflect central molecular alterations. Zilliox et al. (2023) identified 55 differentially expressed blood miRNAs in severe TBI-induced DoC, with six core miRNAs validated by reverse transcription-polymerase chain reaction (RT-PCR) and linked to secondary brain injury pathways, nerve repair processes, neurobehavioral performance, and post-injury duration. Although this RNA-seq study provided an unbiased discovery profile, the very small sample size limits generalizability and increases the risk that identified signatures may be cohort-specific.

Overall, current DoC-related miRNA evidence suggests that circulating miRNAs are more suitable for subgroup stratification than for standalone diagnosis (Musso et al., 2023; Petrova et al., 2022; Zilliox et al., 2023). Existing findings indicate that miRNA profiles may be influenced by etiology, post-injury time, and recovery status, but it remains unclear whether they capture impaired consciousness itself or broader injury-related regulatory responses. Therefore, future studies should move from single-miRNA discovery toward predefined multi-miRNA panels tested in etiologically and temporally stratified DoC cohorts, with evaluation of their incremental value beyond established clinical and multimodal assessments.

These findings indicate that circulating miRNAs may be more useful for capturing etiological and temporal heterogeneity than for serving as standalone diagnostic markers of impaired consciousness. Because a single miRNA may capture only one component of injury response, inflammation, repair, or systemic regulation, future studies should prioritize predefined multi-miRNA panels combined with clinical and neurophysiological variables. These panels should be tested in etiologically and temporally stratified DoC cohorts, including TBI-related DoC, HIBI-related DoC, chronic UWS, MCS, MCS+, and recovery-stage patients. Standardization of specimen selection, reference controls, qRT-PCR or dPCR conditions, and independent external validation will be essential before miRNA signatures can be evaluated as clinically meaningful tools for DoC stratification.

#### 2.3.2 Exosomal miRNA in peripheral blood

Exosomal miRNAs may offer improved stability and biological specificity compared with free circulating miRNAs because they are protected within vesicles and may partially reflect intercellular communication after brain injury. In TBI research, serum exosomal miRNAs such as miR-206 and miR-549a-3p have been associated with classical injury-related biomarkers, including BDNF, NSE, and S100B (Yang et al., 2024). This suggests that exosomal miRNAs may capture injury-related molecular responses and may complement protein biomarkers. However, direct evidence in DoC is currently lacking, and it remains unclear whether exosomal miRNA profiles provide information beyond conventional circulating miRNAs or injury-related protein markers.

Overall, circulating and exosomal miRNA biomarkers remain promising but immature. Key barriers include inconsistent detection protocols, variable internal reference genes, heterogeneous analytical pipelines, and limited large-scale multicenter validation. Strategies such as multi-miRNA combination models and longitudinal dynamic monitoring developed in TBI research may provide useful references, but DoC-specific validation in etiology- and stage-stratified cohorts remains essential before clinical translation (Di Pietro et al., 2017; Musso et al., 2023; Sessa et al., 2025).

## 3 Cerebrospinal fluid biomarkers

**Figure 5:**
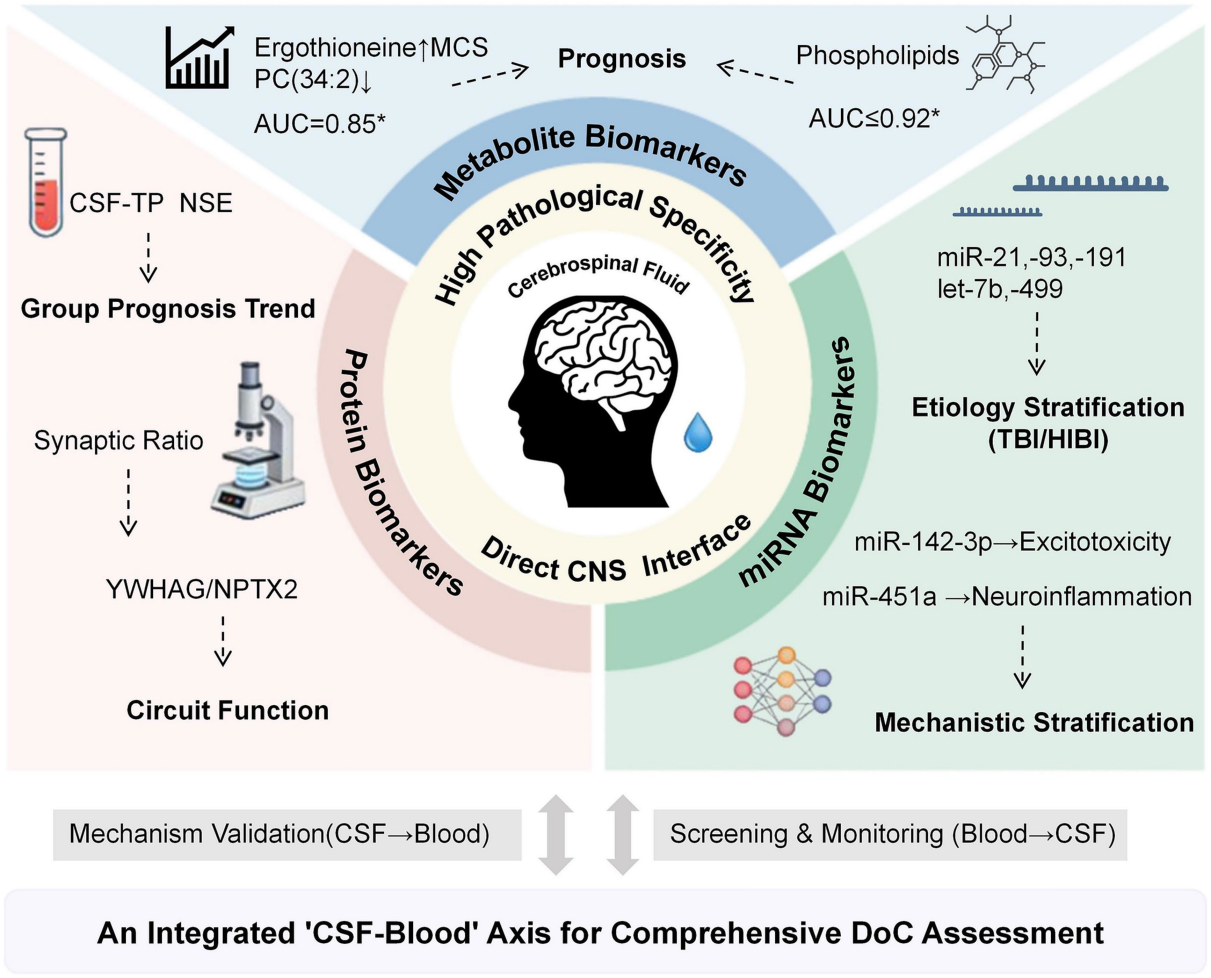
Cerebrospinal fluid biomarkers within a peripheral–central assessment framework for disorders of consciousness. This schematic illustrates an integrated CSF–blood axis for comprehensive disorders of consciousness (DoC) assessment. CSF biomarkers are organized into three categories: protein biomarkers, metabolite biomarkers, and miRNA biomarkers, reflecting the high pathological specificity and direct CNS interface of cerebrospinal fluid. Protein biomarkers include CSF-TP and NSE, which are linked to group prognosis trends, and YWHAG/NPTX2, which are linked to circuit function through synaptic ratio assessment. Metabolite biomarkers include ergothioneine, PC(34:2), and phospholipids, which are associated with prognosis. miRNA biomarkers include miR-21, miR-93, miR-191, let-7b, and miR-499 for etiology stratification between TBI and HIBI, as well as miR-142-3p and miR-451a for mechanistic stratification related to excitotoxicity and neuroinflammation. The lower panel presents a bidirectional CSF–blood framework, in which CSF supports mechanism validation and blood supports screening and monitoring.

While peripheral blood biomarkers excel in non-invasive screening, prognostic prediction, and dynamic monitoring due to their accessibility, they are inherently limited by insufficient CNS-specificity, as systemic physiological changes can confound their interpretation. In contrast, CSF, as a biofluid in direct communication with the CNS, provides a more central information layer for assessing pathological processes such as neuroinflammation, synaptic dysfunction, and metabolic disturbance. Therefore, CSF biomarkers may complement blood-based markers within a “peripheral–central” integrated assessment framework for DoC (Figure 5; Wang et al., 2025; Petrova et al., 2022; Xu et al., 2023; Liu et al., 2024; Liu et al., 2023; De Vito et al., 2023; Feng et al., 2023). However, their clinical role should be interpreted cautiously: CSF biomarkers are more suitable for mechanistic clarification, selected differential diagnosis, and central validation of peripheral findings than for routine screening or long-term repeated monitoring.

### 3.1 Protein biomarkers in CSF

CSF protein biomarkers are more directly exposed to the central microenvironment than peripheral blood markers and may therefore provide information on CNS injury, synaptic dysfunction, and central inflammatory responses in DoC. Methodologies in CSF protein biomarker research vary from routine clinical assays to sophisticated multi-omics approaches, reflecting a spectrum from clinically accessible injury markers to mechanism-oriented discovery tools.

Early clinical studies primarily relied on easily detectable but relatively non-specific injury markers. For example, Liu et al. (2024) and Liu et al. (2023) analyzed CSF total protein (CSF-TP) using the pyrogallol red molybdate method and NSE using ELISA, and found that both markers were significantly elevated in VS compared with MCS patients, with combined detection showing prognostic value. However, CSF-TP is a highly non-specific marker and can be confounded by complications such as infection or hemorrhage. Although NSE has some neuronal relevance, its release kinetics post-acute injury are complex, and it can be influenced by extracranial injury. Therefore, these markers are more suitable for identifying group-level injury-related trends than for precise individual diagnosis or mechanistic interpretation of impaired consciousness.

More recent studies have used high-throughput proteomics to identify CSF markers more closely related to synaptic integrity and consciousness-relevant neural function. For example, Oh et al. identified the CSF YWHAG/NPTX2 synaptic protein ratio as a predictor of cognitive outcome using SomaScan, liquid chromatography–tandem mass spectrometry (LC–MS/MS), and machine-learning analyses (Oh et al., 2025). Compared with broad injury markers such as CSF-TP or NSE, such synaptic protein signatures may provide information beyond general injury burden by reflecting synapse-related dysfunction and outcome-associated neural changes. However, the complexity, cost, and technical requirements of such approaches currently limit their routine clinical application.

Compared with blood samples, CSF protein biomarkers have important practical limitations. CSF collection is typically invasive, poorly tolerated by patients, and consequently unsuitable for long-term dynamic monitoring. Some biomarkers lack specificity and can be confounded by factors such as infection, hemorrhage, or other complications. In contrast, serum detection of proteins such as GFAP and S100B is less invasive and has broader clinical applicability in TBI contexts (Di Pietro et al., 2017).

### 3.2 Metabolic alterations in CSF

**Figure 6:**
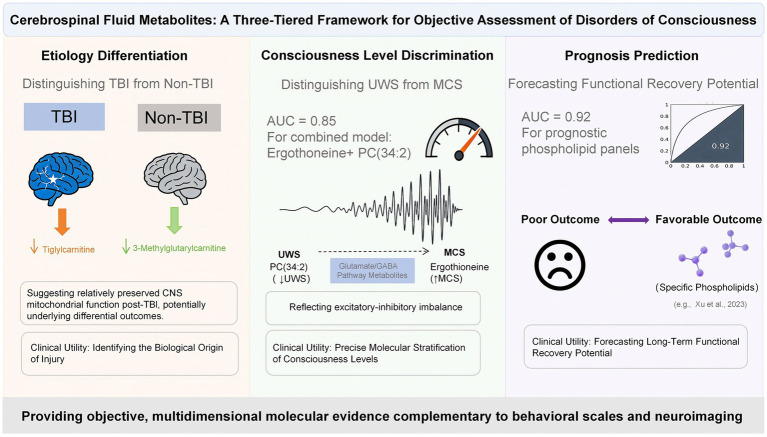
A three-tiered diagnostic framework of CSF metabolites in disorders of consciousness. This schematic summarizes the potential role of cerebrospinal fluid (CSF) metabolites in three assessment domains for disorders of consciousness (DoC): etiological differentiation, consciousness-level discrimination, and prognosis prediction. In etiological differentiation, specific metabolic changes may help distinguish traumatic brain injury (TBI)-related DoC from non-TBI DoC and may reflect differences in central energy or mitochondrial-related metabolic states. In consciousness-level discrimination, combined CSF metabolite signatures, such as ergothioneine and PC (34:2), may help distinguish unresponsive wakefulness syndrome (UWS) from minimally conscious state (MCS), potentially reflecting excitatory–inhibitory imbalance and pathway-level metabolic alterations. In prognosis prediction, phospholipid-related metabolite panels may be associated with long-term functional recovery potential. Overall, CSF metabolomics may provide multidimensional molecular information complementary to behavioral scales and neuroimaging, but current evidence remains exploratory and requires independent validation before clinical application.

CSF metabolomics focuses on local metabolic disturbances in the CNS, enabling the identification of dysregulation in core pathological pathways within DoC brains (Figure 6). However, current evidence remains exploratory, with findings mainly derived from small cohorts, untargeted platforms, and multivariate discovery models.

Recent studies suggest that CSF metabolic signatures may contribute to consciousness-level differentiation and outcome prediction. Wang et al. identified 14 differential CSF metabolites in DoC patients, mainly involving necrosis/apoptosis, amino acid metabolism, and neuroprotection-related pathways. Ergothioneine was significantly upregulated in MCS patients, while PC (34:2) was negatively correlated with brain injury severity. A combined CSF panel of ergothioneine and PC (34:2) achieved an AUC of 0.85 for distinguishing UWS from MCS (Wang et al., 2025; Figure 4A). Similarly, Xu et al. (2023) used LC–MS-based CSF metabolomics in 51 DoC patients and reported etiology-related acylcarnitine profiles, consciousness-level-related glutamate–GABA pathway metabolites, and prognosis-associated phospholipids, with a reported AUC of up to 0.92. These findings indicate that CSF metabolites may capture central metabolic alterations related to etiology, consciousness level, and prognosis. Nevertheless, their clinical readiness remains limited because most candidate panels have not yet undergone independent external validation.

Mechanistically, CSF metabolic alterations may reflect central processes relevant to DoC, including amino acid neurotransmission, energy metabolism, phospholipid remodeling, neuroinflammation, and blood–brain barrier dysfunction (Migdady et al., 2025). However, whether these signatures specifically reflect impaired consciousness or broader CNS injury, disease stage, and secondary complications remains uncertain. From a translational perspective, CSF metabolomics is better positioned for mechanism exploration, target discovery, and central validation of peripheral findings than for routine screening. In Wang et al.’s (2025) study, serum metabolomics showed higher diagnostic performance and greater feasibility for repeated testing than CSF metabolomics, supporting a complementary rather than competing role for CSF-based metabolic analysis.

Although LC–MS-based untargeted platforms are powerful tools for detecting CSF metabolic alterations, their outputs remain sensitive to peak picking, alignment, normalization, quality control, metabolite identification, and multivariate modeling. Therefore, future studies should establish consensus CSF metabolomics workflows and validate predefined metabolite panels in multicenter DoC cohorts stratified by etiology, consciousness level, disease stage, and outcome.

### 3.3 MiRNA biomarkers in CSF

As central-specific regulatory molecules, CSF miRNAs may provide information on intracerebral gene-regulatory changes relevant to DoC etiological stratification and pathological mechanism research. However, their current role remains exploratory, and direct clinical applicability requires further validation.

Studies have reported significant differences in the expression of miR-21, miR-93, miR-191, let-7b, and miR-499 in the CSF of patients with pDoC, with potential value for distinguishing TBI-related DoC from HIBI-related DoC (Petrova et al., 2022). These studies used TaqMan probe-based quantitative polymerase chain reaction (qPCR) and introduced exogenous cel-miR-39 for normalization. These findings suggest that CSF miRNAs may complement blood miRNAs by providing a more CNS-proximal regulatory signal for etiological stratification. However, the study was hypothesis-driven and tested miRNAs already known to be associated with brain injury, which may have missed novel DoC-specific candidates. The relatively small sample size also limits statistical power and generalizability.

Some experimental studies have further explored the functional roles of specific miRNAs within the CNS. For example, studies of miR-142-3p and miR-451a suggest that these miRNAs may be involved in excitotoxicity or neuroinflammatory regulation through pathways such as astrocytic glutamate transporters and TLR4/NF-κB signaling (De Vito et al., 2023; Feng et al., 2023). These studies provide mechanistic hypotheses, but much of the evidence comes from animal models or cell experiments rather than direct validation in DoC cohorts. Therefore, these miRNAs should be regarded as biologically plausible candidates rather than established CSF biomarkers for DoC.

In summary, CSF protein, metabolite, and miRNA biomarkers collectively form a central information layer for mechanistic clarification, etiological differentiation, target discovery, and selected differential diagnosis in DoC, especially when integrated with peripheral biomarkers in a dual-biofluid strategy. However, current CSF findings remain heterogeneous, ranging from low-specificity routine injury markers to more mechanism-oriented proteomic or molecular candidates that still require independent validation.

From a broader clinical perspective, CSF biomarkers are unlikely to replace blood-based biomarkers because CSF collection is invasive, poorly suited for repeated monitoring, and may be difficult to implement in chronically bedridden or clinically unstable DoC patients. Lumbar puncture may also be limited by contraindications or complications such as coagulation abnormalities, spinal injury, intracranial hypertension, post-puncture headache, bleeding, CSF leakage, or infection (Engelborghs et al., 2017; Reis et al., 2024). Therefore, CSF biomarkers are better positioned as selective adjunctive tools for mechanism confirmation, central validation, or differential diagnosis, whereas peripheral blood biomarkers remain more feasible for longitudinal monitoring, large-scale studies, and future clinical implementation.

## 4 Fecal biomarkers

**Figure 7:**
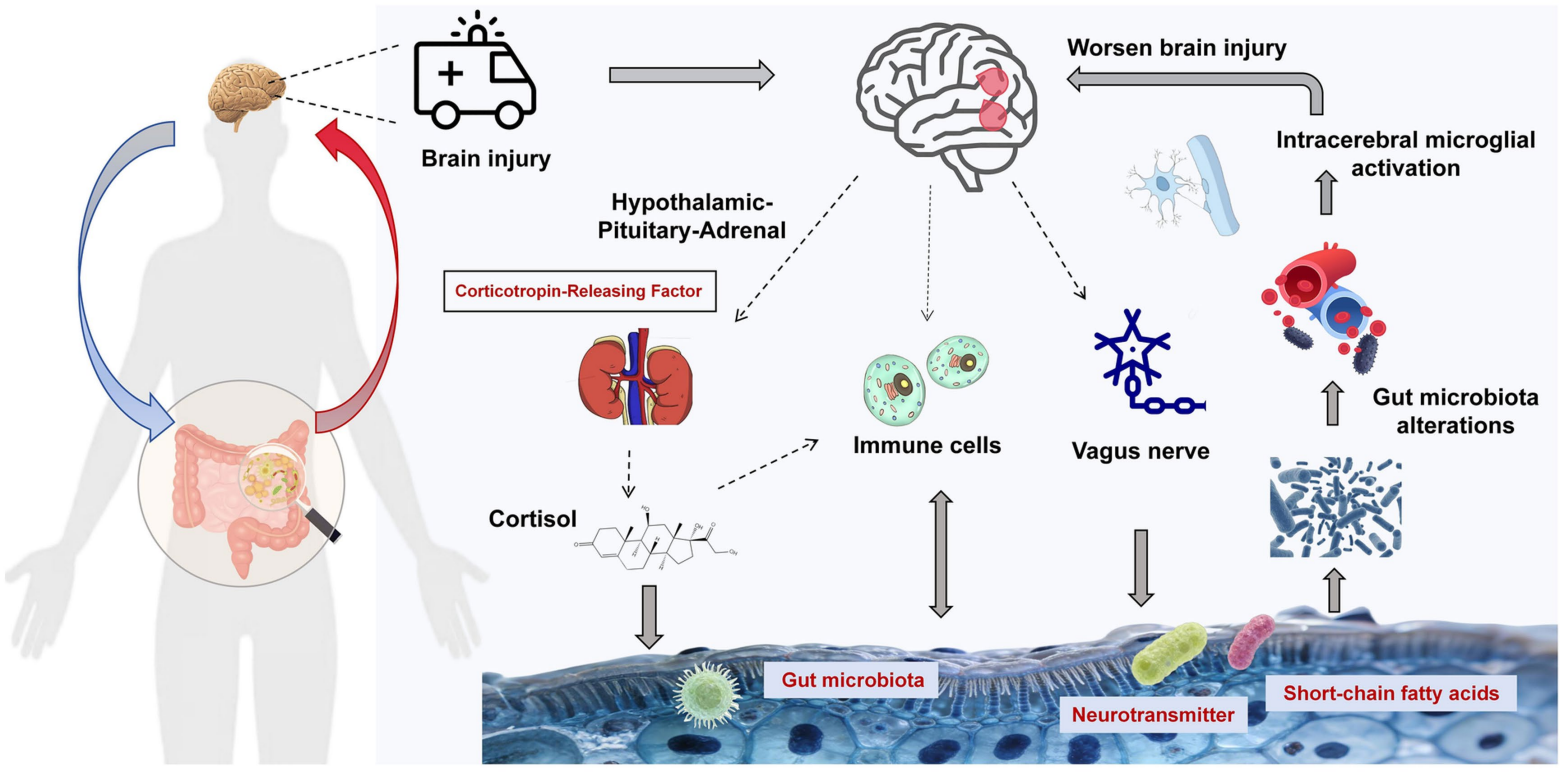
Schematic illustration of the GBA and its proposed mechanisms in disorders of consciousness. This schematic illustrates the potential bidirectional communication between brain injury-related disorders of consciousness (DoC) and gut microbiota alterations. Brain injury may affect gut ecology through the hypothalamic–pituitary–adrenal (HPA) axis, corticotropin-releasing factor (CRF), cortisol release, immune-cell modulation, and vagus nerve/autonomic pathways. Cortisol may further influence immune-cell activity, inflammatory responses, gut barrier function, and microbial composition. Conversely, gut microbiota alterations and related products, including SCFAs and neurotransmitters, may be linked to intracerebral microglial activation and worsening brain injury.

Building on the central information provided by CSF biomarkers and the clinical accessibility of blood biomarkers, fecal biomarkers have emerged as an exploratory frontier in DoC research through the gut–brain axis (GBA). Fecal biomarkers, encompassing gut microbiota and their metabolites, may provide insight into potential intervention-related targets by reflecting gut-brain axis-related changes associated with neural, immune, and endocrine pathways, thereby extending the “central-peripheral-intestinal” multimodal framework (Figure 7).

In pDoC patients, preliminary studies have suggested associations between gut dysbiosis, microbial metabolites, electrophysiological features, and consciousness levels. However, this field remains at an early exploratory stage. Current evidence is mainly derived from small-sample, single-center studies, preclinical models, and indirect findings from related neurological or systemic disorders. Therefore, fecal biomarkers should currently be interpreted as hypothesis-generating tools for understanding systemic contributions to DoC pathophysiology rather than clinically established diagnostic or prognostic biomarkers.

### 4.1 The gut-brain axis

In recent years, the GBA has attracted increasing attention in neuroscience because gut microbiota can communicate bidirectionally with the brain through neural, immune, endocrine, serotonergic, and tryptophan-related pathways. Microbiota-derived metabolites, such as short-chain fatty acids (SCFAs), trimethylamine-N-oxide, and indole derivatives, may influence glial function, neuroinflammation, and blood–brain barrier integrity (Dalal et al., 2025; Ghaemi and Kheradmand, 2025). Research by Chen et al. (2025b) summarized five GBA signaling pathways, including neural, immune, enteroendocrine, serotonin, and tryptophan pathways, and suggested that interventions such as acupuncture may modulate autonomic tone, cholinergic anti-inflammatory pathways, neuroglial function, and tryptophan–serotonin metabolism in TBI-related contexts. These findings provide a biological rationale for exploring the GBA in DoC, particularly because TBI and other severe brain injuries may disrupt intestinal homeostasis and systemic inflammatory regulation. However, GBA-related evidence in DoC remains limited.

Although preliminary studies have reported gut microbiota dysbiosis and metabolic disturbances in pDoC patients, biomarker studies specifically focusing on gut microbiota and metabolomics in DoC remain extremely limited. Therefore, GBA-related findings should be viewed as a mechanistic framework for hypothesis generation rather than evidence of clinically actionable fecal biomarkers.

### 4.2 Fecal proteomics

Protein molecules involved in GBA-related signaling may represent potential targets for exploratory fecal proteomics research in DoC. A foundational animal study by Wang et al. employed a controlled cortical impact model with 16S rDNA sequencing and untargeted LC–MS metabolomics (Wang et al., 2021). Using multivariate statistics and correlation analysis, they reported associations consistent with a potential “brain injury - gut dysbiosis - neuroinflammation” interaction. This provides preclinical evidence suggesting that GBA disruption may contribute to secondary injury and may involve inflammation-related proteins as mechanistic links.

This is supported indirectly by clinical trials in related disorders. Bajaj et al. conducted randomized controlled trials in patients with cirrhosis and hepatic encephalopathy (HE) using 16S rRNA sequencing, LC/MS-based bile acid profiling, ELISA detection of inflammatory proteins such as IL-6 and lipopolysaccharide-binding protein (LBP), and cognitive assessment with EncephalApp. Their findings suggested that fecal microbiota transplantation (FMT) capsules enriched with specific bacterial families were safe and could reduce serum inflammatory proteins, with associations with improved cognitive performance (Bajaj et al., 2019). Although these findings are not directly applicable to DoC, they suggest that gut-ecology modulation may influence systemic inflammatory pathways that are conceptually relevant to DoC research.

However, fecal proteomic or inflammation-related biomarkers in DoC remain largely conceptual. Future studies should determine whether fecal or serum inflammation-related protein signals correlate with DoC-specific neural injury, glial activation, consciousness level, and recovery trajectory, rather than merely reflecting systemic inflammation or comorbid medical conditions. Large-sample clinical studies in well-characterized DoC cohorts are needed before these markers can be considered translationally meaningful.

### 4.3 Fecal metabolomics

**Figure 8:**
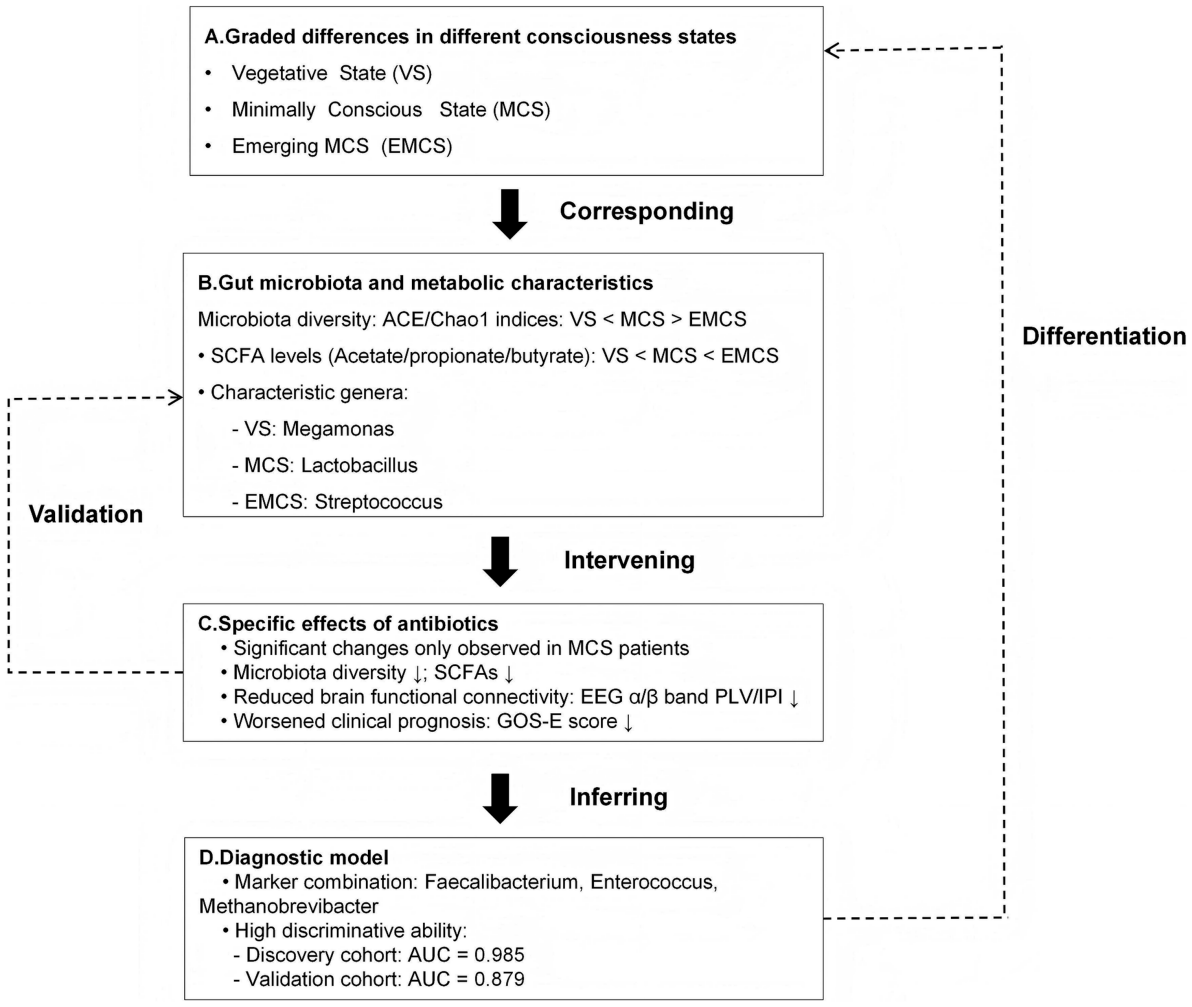
Gut microbiota signatures in prolonged disorders of consciousness. This schematic illustrates reported relationships among consciousness levels, gut microbiota characteristics, antibiotic-related changes, and microbial biomarker models in prolonged disorders of consciousness (pDoC; Yu et al., 2022). The figure includes graded consciousness states, including vegetative state/unresponsive wakefulness syndrome (VS/UWS), minimally conscious state (MCS), and emergence from minimally conscious state (EMCS). It also presents differences in microbiota diversity, short-chain fatty acid (SCFA) levels, and characteristic genera across these states. Antibiotic-related changes are shown as changes in microbiota diversity and SCFA levels, reduced EEG functional connectivity, and lower GOS-E score. A diagnostic model based on Faecalibacterium, Enterococcus, and Methanobrevibacter is shown, with AUC values.

As principal gut microbiota metabolites, SCFAs are pivotal molecular mediators of GBA communication. A pioneering study by Yu et al. (2022) systematically reported gradient differences in gut microbiota structure and SCFA metabolism among VS, MCS, and EMCS patients, with SCFA levels increasing in parallel with improved consciousness status. This case–control study used 16S rRNA sequencing, targeted gas chromatography–mass spectrometry (GC–MS) for SCFA detection, and EEG functional connectivity analysis to develop a diagnostic model based on microbial metabolic features. Crucially, they found that antibiotic intervention specifically reduced gut microbiota diversity and SCFA levels in MCS patients, a change positively correlated with weakened EEG connectivity and poor clinical prognosis. This work provides preliminary clinical evidence suggesting associations between GBA-related metabolic alterations, electrophysiological connectivity, and clinical status in DoC (Figure 8). Furthermore, narrative reviews of heterogeneous FMT trials in other cognitive disorders suggest that FMT may modify gut ecology and cognition-related metabolic pathways, with microbiota and metabolite changes reported to correlate with cognitive outcomes (Alaeddin et al., 2025; Chen et al., 2023; Ren et al., 2025). However, these studies have not directly targeted DoC patients, and their applicability to DoC remains speculative. Dedicated clinical trials in DoC cohorts are needed before FMT or other GBA-targeted interventions can be considered clinically relevant for consciousness recovery.

From a methodological perspective, fecal metabolomics requires substantial standardization. The combination of 16S rRNA sequencing and targeted GC–MS for SCFA analysis represents a focused approach for assessing microbiota composition and predefined microbial metabolites. Untargeted metabolomics could serve as a complementary strategy to uncover a broader spectrum of relevant gut-derived metabolites. To advance this field, standardized protocols encompassing sample collection, storage, DNA/metabolite extraction methods, and bioinformatic pipelines for 16S rRNA data analysis are urgently needed to ensure data comparability.

Collectively, fecal biomarkers and gut-brain axis-related signatures represent a conceptually important but still exploratory frontier in DoC research. Preliminary findings suggest associations among gut microbiota composition, SCFA metabolism, inflammatory pathways, electrophysiological connectivity, and consciousness levels, but they do not yet establish causal mechanisms or clinically actionable biomarkers. Fecal microbiota and metabolite profiles are also highly susceptible to diet, enteral nutrition, antibiotics, medications, infection, gastrointestinal function, and other systemic factors, which substantially limit reproducibility and disease specificity. Therefore, at present, this field should be regarded as exploratory rather than clinically informative. Fecal biomarkers are better positioned as hypothesis-generating tools for investigating systemic contributions to DoC pathophysiology and potential intervention-related targets, rather than as biomarkers for diagnosis, prognosis, or treatment decision-making. Future studies should employ prospective multicenter designs, standardized stool collection and storage protocols, controlled nutritional and medication records, longitudinal sampling, and integrated analyses with clinical, electrophysiological, neuroimaging, and multi-biofluid data.

## 5 Biomarkers from other non-invasive sample sources

Besides core biomarkers derived from CSF, serum, and feces, urine and saliva represent supplementary non-invasive sample sources for DoC biomarker research because of their low sampling burden and feasibility for repeated collection.

### 5.1 Biomarkers from urine

As a fully non-invasive sample, urine may support low-burden longitudinal monitoring in DoC, but current evidence remains exploratory.

A comprehensive untargeted metabolomics study by Wang et al. (2025) analyzed CSF, serum, and urine from DoC patients. In urine, 22 differential metabolites, including 19 endogenous metabolites, were identified between UWS and MCS, mainly involving cell signaling, neural regulation, and oxidative stress. A diagnostic panel comprising 5-methoxyindoleacetate and cis-4-hydroxycyclohexylacetic acid, both associated with tyrosine metabolism, achieved an AUC of 0.93 for distinguishing UWS from MCS (Figure 4A). These findings suggest that urinary metabolomics may capture systemic metabolic perturbations associated with consciousness status, although their biological interpretation remains uncertain.

Urine also shows exploratory value in etiological differentiation of DoC. A multi-biofluid UPLC-MS study of traumatic and non-traumatic DoC patients reported distinct metabolite panels across CSF, serum, and urine, with a urinary panel composed of dihydropetasin lactone and doxepin-N-oxide glucuronide achieving an AUC of 1.000 for etiological differentiation (Xiao et al., 2024). However, such very high diagnostic performance in small or exploratory cohorts should be interpreted cautiously because high-dimensional biomarker models are susceptible to overfitting, internal-validation bias, cohort heterogeneity, and pre-analytical or analytical variability. Without independent external validation in larger multicenter DoC cohorts, these AUC values should be regarded as preliminary indicators of discriminatory potential rather than evidence of clinical readiness.

Urinary biomarkers have several inherent limitations that complicate their interpretation in DoC. Urinary metabolite and protein levels can be influenced by renal clearance, tubular reabsorption and secretion, urine dilution, hydration status, systemic metabolism, diet, medication exposure, physical activity, and comorbid diseases (Seol et al., 2020; Stevens et al., 2019). Moreover, CNS-derived molecules must pass through the blood–brain barrier, systemic circulation, and renal filtration before being detected in urine, during which they may be diluted, degraded, reabsorbed, or cleared at very low concentrations. Renal function may further influence circulating-to-urinary biomarker clearance, supporting the need to record and adjust for kidney function, hydration status, sampling time, urine concentration, and normalization strategy (Yuan et al., 2025). Common correction methods such as creatinine normalization may also be affected by kidney function, muscle mass, age, nutritional status, and systemic illness (Baxmann et al., 2008; Kuiper et al., 2021). Pre-analytical variables, including urine collection time, storage conditions, freeze–thaw cycles, and data-processing workflows, may further affect metabolomic reproducibility (Stevens et al., 2019; Laparre et al., 2017). Therefore, urinary biomarkers remain exploratory candidates for low-burden longitudinal monitoring in selected stable patients rather than standalone diagnostic or prognostic tools, and their specificity should be validated against neurological, renal, inflammatory, and metabolic conditions.

### 5.2 Biomarkers from saliva

Saliva forms a functional association with the CNS through the “brain-saliva axis” (Sansores-España et al., 2021; Zürcher and Humpel, 2023). It contains miRNA, proteins, metabolites, and oral microbiota, which may provide indirect information on neural injury, systemic responses, and repair-related processes. The non-invasive and low-burden nature of saliva collection makes it theoretically attractive for future studies involving patients with limited ability to cooperate with conventional sampling procedures, including pediatric patients and severely impaired DoC patients.

Current evidence for salivary biomarkers in DoC is still very limited, and most support comes from TBI or neurodegenerative disease research. In TBI, a systematic review identified 188 differentially expressed salivary miRNAs, with 30, including miR-133a and miR-206, replicated across studies (Hiskens et al., 2022). This suggests a degree of consistency in the salivary miRNA response to brain injury, supporting its exploration in DoC. Direct evidence in DoC is beginning to emerge. A pioneering case–control study by Xu et al. employed 16S rRNA sequencing of oral swabs from pDoC patients. They found significantly altered oral microbiomes, with reduced β-diversity in VS/UWS and specific bacterial taxa showing correlations with consciousness levels (Xu et al., 2025). This study provides early evidence that oral microbiome alterations accessible through saliva or oral swabs may be associated with consciousness status in DoC. However, these findings remain preliminary and require validation in larger longitudinal cohorts. Salivary research in established neurodegenerative diseases provides additional indirect support. Salivary studies in neurodegenerative diseases further support the broader principle that saliva can contain CNS-relevant pathological proteins, such as Aβ/tau-related markers in Alzheimer’s disease and α-synuclein in Parkinson’s disease, but their relevance to DoC remains indirect and requires dedicated validation (Boschi et al., 2022; Pawlik and Błochowiak, 2021).

The main advantage of saliva lies in its high accessibility and low-burden sampling, which may facilitate repeated specimen collection in future longitudinal studies. However, its translational value in DoC remains uncertain. Salivary biomarkers are susceptible to diet, oral hygiene status, local inflammation, and drugs, leading to fluctuations in the concentration of target biomarkers. Moreover, salivary signals may reflect oral microbial ecology, systemic inflammation, or peripheral stress rather than CNS-specific pathology, limiting disease specificity. Current evidence remains fragmented. Studies differ in target analytes, sampling methods, analytical platforms, research objectives, and evidence levels, and no unified multi-analyte salivary signature has been established for DoC. Therefore, TBI-derived salivary miRNA candidates such as miR-133a should be regarded as indirect exploratory references rather than validated DoC biomarkers. Future studies should establish standardized collection and analytical protocols, evaluate DoC-specific salivary biomarker profiles in large longitudinal cohorts, and determine whether saliva provides complementary value to blood-based and urine-based markers within multi-biofluid frameworks.

## 6 Discussion

Previous biomarker studies in this field have primarily focused on acute TBI, single biofluid sources, or individual omics platforms. This fragmented evidence base has limited consistent interpretation of biomarker findings within the clinically heterogeneous context of DoC. In the present review, multi-specimen biomarker evidence is therefore considered within an integrated translational framework that emphasizes biological proximity, disease specificity, evidence maturity, and clinical applicability. Within this framework, the clinical relevance of a biomarker is not defined solely by its statistical association with diagnostic or prognostic outcomes, but also by its capacity to reflect relevant pathological processes, distinguish impaired consciousness from general brain injury responses, and provide interpretable information beyond established behavioral, electrophysiological, and neuroimaging assessments. This perspective provides the basis for a critical synthesis of multi-specimen evidence, recurrent biological pathways, biomarker panels with translational potential, patient subgroup stratification, and the methodological requirements needed for clinical translation.

### 6.1 Interpretation of multi-specimen biomarker evidence

The available evidence indicates that biomarkers identified in blood, CSF, feces, urine, and saliva should be interpreted within a broad central–peripheral–intestinal pattern rather than as parallel molecular findings across different biofluids. Blood-based biomarkers may provide accessible information on systemic injury responses and longitudinal changes, CSF biomarkers may offer CNS-proximal mechanistic information, and fecal, urinary, and salivary biomarkers currently remain more indirect indicators of systemic regulation. However, the translational relevance of these specimen sources remains uneven. Some markers primarily reflect injury severity (Papa et al., 2014; Mondello et al., 2012), whereas markers related to axonal integrity, synaptic function, or outcome-associated neural changes may be more closely linked to network integrity or recovery potential (Bagnato et al., 2021; Oh et al., 2025).

Although TBI biomarker research provides an important methodological foundation, particularly for blood-based injury markers, direct extrapolation from acute TBI to pDoC, non-traumatic etiologies, or mixed injury mechanisms remains limited. The biological determinants of persistent impaired consciousness are not identical to those of acute injury severity. Therefore, DoC biomarker evidence should be interpreted according to etiology, disease stage, consciousness level, sampling window, and validation status. Within this interpretive framework, Table 1 summarizes biomarker findings according to specimen source, and Table 2 presents the evidence status, methodological characteristics, validation stage, and translational relevance of the included studies.

**Table 1:** Summary of biomarkers across different sources.

Sample type	Sample groups	Main biomarkers	Key methods	Main findings	References
Serum	pDoC patients (TBI/HIBI etiology)	NFL	Simoa	NFL levels correlated with consciousness impairment early and with etiology later.	Bagnato et al. (2021)
Serum	Amateur boxers vs. healthy controls	NSE	Biochip array technology (evidence Investigator platform)	Serum NSE levels were significantly higher in boxers (median: 11 ng/mL) than in controls (4.8 ng/mL; *p* = 0.014) and remained elevated after a 2-month rest period.	Zetterberg et al. (2009)
Serum	Trauma patients (with MMTBI vs. without MMTBI, ±extracranial fractures)	GFAP, S100B	ELISA	GFAP demonstrated higher specificity than S100B (affected by extracranial fractures) in detecting TBI intracranial lesions	Papa et al. (2014)
Serum	DoC patients	GFAP, NFL	Simoa	GFAP was associated with white matter microstructural damage on neuroimaging.	Coppola et al. (2022)
Serum	mTBI patients vs. healthy controls	CRP, MMP-2, CKBB, miR-16, miR-92a	ELISA, CLIA, qRT-PCR, miRNA-seq, TLDA	The combined panel predicts mild TBI positive CT (NPV = 97.2, c-statistic = 0.975), analogous to the role of troponin-I in myocardial infarction	Al-Adli et al. (2021)
Serum/CSF	sTBI patients vs. healthy controls	UCH-L1	Sandwich ELISA	UCH-L1 was elevated within 6 h, remained high for 7d, and predicted 3-month mortality at a cutoff of >5.22 ng/mL (OR = 4.8). AUCs were significant (*p* < 0.001). Distinguished mild from severe TBI	Mondello et al. (2012)
Rat brain tissue (peri-contusional critical zone, contralateral uninjured zone)	sTBI rats (24 h/72 h/120 h/1w/2w/3w; 8 rats/group) vs. sham control (craniotomy without impact)	TNF-α, IL-6	Free-fall impact; immunohistochemical staining (SP); quantitative gray value analysis	TNF-α expression peaked early (peak 3-5d) in secondary injury/repair; IL-6 peaks at 1w (TNF-α-induced) for repair; expression peaks align with cerebral edema, CSF indirectly reflects intracranial inflammation	Yu (2008)
Plasma, CSF, contused brain tissue	sTBI; mTBI vs. healthy controls	RANTES	ELISA	Plasma RANTES was higher in TBI vs. controls (correlates with severity), predicts severe TBI early mortality, and exceeds CSF/tissue levels (systemic inflammation involved)	Albert et al. (2017)
Plasma	DoC patients	IL-13, TNF-α	ELISA	Correlated with CRS-R scores and independently predicted 12-month functional recovery.	Wu et al. (2021)
Serum	DoC patients	ACTH	CLIA	Higher serum ACTH was an independent protective predictor of consciousness recovery at 6 months	Wu et al. (2024)
Serum	TBI DoC patients vs. healthy controls	53 differential metabolites (creatine, carnitine, glutamate)	UHPLC-Q-Orbitrap HRMS	24 upregulated/29 downregulated metabolites identified; 10 key metabolic pathways linked to DoC pathogenesis.	Feng et al. (2024)
Serum, CSF, urine	UWS vs. MCS patients	Various metabolites (LysoSM, LysoPC, bile acid derivatives)	UPLC–MS/MS	A serum metabolite panel achieved high diagnostic accuracy (AUC = 0.94) for distinguishing UWS from MCS.	Wang et al. (2025)
Serum	DoC patients	Purine metabolites, phospholipids, arachidonic acid	Targeted metabolomics (LC–MS/MS, GC–MS)	Purine metabolism was significantly suppressed; specific metabolites correlated positively with CRS-R scores.	Yu et al. (2021)
Serum	DoC patients vs. healthy controls	TBIL	Biochemical assay (total nitrogen method)	Elevated serum TBIL independently predicted a favorable prognosis at 1, 3, and 6 months; tertiles show a positive “good outcome” trend, consistent across subpopulations.	Huang et al. (2024)
Serum	TBI vs. non-TBI DoC Patients	Glycerophospholipids, energy metabolism-related metabolites	LC–MS	Revealed distinct metabolic profiles across etiology, consciousness level, and prognosis	Ge et al. (2024)
Serum	TBI patients vs. healthy controls	miR-93, miR-191, miR-499	qRT-PCR	miR-93 distinguished TBI from controls with an AUC of 1.000 (internal validation only)	Yang et al., (2016)
Serum	TBI patients (different time points)	miR-425-5p, miR-21	qRT-PCR	miR-425-5p correlated with 6-month prognosis; miR-21 was prominent for prognosis prediction at 4–12 h post-injury	Di Pietro et al. (2017)
Plasma	sTBI patients	miR-142-3p, miR-423-3p	qRT-PCR	Expression altered within 6 h post-trauma, enabling effective prediction of sTBI	Schindler et al. (2020)
Serum	mTBI vs. sTBI patients	miR-219a-5p	TLDA, dPCR	Could distinguish mTBI from sTBI patients; negatively correlated with 6-month GOS score	Yan et al. (2019)
Serum	DoC patients (TBI/HIBI) vs. healthy controls	miR-150-5p, miR-132-3p, miR-23b-3p, miR-451a, miR-16-5p	dPCR	miRNA expression patterns were etiology- and time-dependent, showing prognostic value.	Musso et al. (2023)
Plasma, CSF	Patients with pDoC	miR-21, miR-93, miR-191, miR-let-7b, miR-499	qRT-PCR	Effectively distinguished DoC caused by TBI from HIBI-induced DoC	Petrova et al. (2022)
Whole blood	DoC patients after sTBI	55 differentially expressed miRNAs	RNA-seq, qRT-PCR	Identified miRNAs associated with secondary injury and neural repair pathways	Zilliox et al. (2023)
Serum exosomes	sTBI/mTBI patients vs. healthy controls; sTBI prognosis subgroups	Exosomal miR-206, miR-549a-3p; BDNF, NSE, S100B	Exosome isolation/characterization (TEM, NTA, WB); qRT-PCR; ELISA	Both miRNAs had diagnostic/prognostic value for TBI; they positively correlated with BDNF, and negatively with NSE/S100B	Yang et al. (2024)
CSF	VS, MCS patients	CSF-TP, NSE	ELISA	CSF-TP and NSE levels were higher in VS; combined detection showed prognostic value	Liu et al. (2024)
CSF	DoC patients (varying etiology/consciousness level)	Acylcarnitines, glutamate-GABA pathway metabolites, phospholipids	LC–MS	Metabolites were valuable for etiological differentiation, consciousness level discrimination, and prognosis prediction	Xu et al. (2023)
CSF, serum	DoC subpopulations (VS/UWS, MCS) vs. healthy controls	Four categories: neuronal (NF-L), glial (GFAP), inflammatory (TNF-α), metabolic (acylcarnitines)	ELISA, Simoa, qRT-PCR, dPCR, LC–MS, RNA-seq,16S rRNA	Combined use of biomarkers with clinical/radiographic data improves individualized DoC diagnosis and prognosis	Migdady et al. (2025)
Feces	VS/UWS, MCS, EMCS patients	SCFAs, gut microbiota	16S rRNA sequencing, targeted GC–MS	SCFA levels increased gradually with improved consciousness and correlated with EEG connectivity and prognosis	Yu et al. (2022)
Urine	TBI-DoC vs. non-TBI-DoC patients	Dihydropetasin lactone, doxepin-N-oxide glucuronide	UPLC-MS	A urinary metabolite panel perfectly discriminated between TBI and non-TBI etiology (AUC = 1.000)	Xiao et al. (2024)
Saliva	TBI patients	188 miRNAs (miR-133a, miR-206)	NGS and nanostring profiling	Salivary miRNAs were differentially expressed in TBI, indicating diagnostic potential	Hiskens et al. (2022)
Saliva	Patients with pDoC	Oral microbiome	16S rRNA sequencing	Oral microbiome diversity and composition were altered and correlated with consciousness levels	Xu et al. (2025)

This table summarizes the sample types, study populations, main biomarkers, analytical methods, principal findings, and references of biomarker studies involving blood, cerebrospinal fluid, feces, urine, saliva, and related biological specimens in DoC and related brain injury populations.

**Table 2:** Evidence status, methodological characteristics, and translational relevance of biomarker studies included in this narrative review.

No.	Biomarker domain/source	Study population/sample size	Study design quality	Evidence level	Validation status	Main methodological limitations or bias risks	Translational positioning	References
1	Serum protein: NfL	pDoC after TBI or HIBI; *n* = 70 patients and 70 age-/sex-matched healthy controls; 52 pts. with 6-month NFL follow-up	Prospective multicenter cohort study	B, 2b	External validation absent	Limited sample size for subgroup comparisons; TBI/HIBI etiological heterogeneity; ELISA sensitivity lower than Simoa; 6-month window may be insufficient for some TBI recovery trajectories	Direct DoC-related prognostic and longitudinal monitoring candidate; serum NFL may reflect sustained axonal degeneration	Bagnato et al. (2021)
2	Blood protein biomarker: NSE	Amateur boxers and healthy controls; *n* = 44 boxers and 23 controls	Observational case–control study	B, 3b	Not validated in DoC	Not DoC-specific; repetitive head trauma model; age difference between groups; limited consciousness-related relevance; no longitudinal outcome validation	Indirect neuronal injury reference; serum NSE may reflect sustained neuronal injury after repetitive head trauma	Zetterberg et al. (2009)
3	Blood protein biomarkers: GFAP, S100B	Adult trauma patients with/without MMTBI; *n* = 397 total, including 209 MMTBI and 188 non-MMTBI trauma patients; 262 underwent head CT	Prospective cohort study	B, 2b	Not validated in DoC	Not DoC-specific; acute trauma cohort; convenience sampling; single-center design; limited extrapolation to pDoC; S100B confounded by extracranial fractures	TBI-related adjunctive diagnostic reference; GFAP more brain-specific than S100B for CT-positive intracranial lesions	Papa et al. (2014)
4	Blood protein biomarkers: GFAP, NFL	pDoC patients and healthy controls; *n* = 16 pDoC patients and 6 healthy controls; 5 pDoC patients had longitudinal follow-up data	Retrospective cohort study	C, 4	External validation absent	Small sample size; mixed etiology; retrospective design; limited longitudinal subgroup; possible diagnostic fluctuation; advanced PET-MRI feasibility limitations	Exploratory multimodal biomarker–imaging framework; NF-L may support longitudinal monitoring, while GFAP may complement imaging-based assessment	Coppola et al. (2022)
5	Blood/serum biomarker evidence across protein, inflammatory, and miRNA categories	TBI biomarker studies across concussion, mild, moderate, severe, and mixed-severity TBI; 115 included articles; 94 distinct biomarkers	Systematic review	B, 2a	Not validated in DoC	Not DoC-specific; TBI-focused evidence; heterogeneous injury severity, outcomes, biomarkers, and study designs; limited direct applicability to pDoC	TBI-related evidence summary and methodological reference for severity-stratified blood biomarker research	Al-Adli et al. (2021)
6	Serum/CSF protein biomarker: UCH-L1	Severe TBI patients and controls; n = 95 severe TBI patients, 167 serum controls, and 24 CSF controls	Prospective case–control study	B, 2b	Not validated in DoC	Not DoC-specific; severe acute TBI only; limited neuroimaging information; GCS-based severity classification; heterogeneous injury mechanisms; limited functional outcome assessment	Indirect neuronal injury and mortality-risk reference; serum/CSF UCH-L1 may support acute TBI diagnosis, severity stratification, and outcome prediction	Mondello et al. (2012)
7	Tissue inflammatory proteins: TNF-α, IL-6	Experimental TBI rat model; *n* = 56 Sprague–Dawley rats, divided into 7 groups with 8 rats per group	Preclinical animal study	D, 5	Preclinical only	Animal model only; tissue-based immunohistochemistry; no human DoC cohort; no external validation; limited clinical outcome relevance	Mechanistic neuroinflammation reference; TNF-α and IL-6 may reflect inflammatory responses after experimental TBI	Yu (2008)
8	Plasma/CSF/tissue inflammatory marker: RANTES/CCL5	Isolated TBI patients and healthy controls; *n* = 70 TBI patients and 15 healthy controls; plasma *n* = 60, CSF *n* = 10, contused brain tissue *n* = 5	Prospective longitudinal case–control study	B, 2b	Not validated in DoC	Not DoC-specific; small CSF and tissue subgroups; CSF, plasma, and tissue samples not from the same patients; GOS assessed at discharge only; infection and systemic inflammation confounding	Indirect neuroinflammation and early mortality-risk reference; plasma RANTES may reflect systemic inflammatory response after TBI	Albert et al. (2017)
9	Blood inflammatory cytokines: IL-13, TNF-α	Patients with pDoC after severe TBI and healthy controls; *n* = 101 pDoC patients and 22 healthy controls	Prospective cohort study	B, 2b	External validation absent	Single-center design; relatively homogeneous sTBI-related DoC cohort; no non-traumatic DoC validation; cytokine non-specificity; infection or systemic inflammation may confound results	Direct DoC-related inflammatory biomarker reference; IL-13 and TNF-α may support prognostic assessment in chronic unconscious phase after sTBI	Wu et al. (2021)
10	Blood neuroendocrine marker: ACTH	Patients with DoC; *n* = 208 recruited, including favorable prognosis subgroup *n* = 38 and poor prognosis subgroup *n* = 156	Retrospective observational study	B, 2b	External validation absent	Retrospective design; single biomarker emphasis; incomplete subgroup total in abstract; potential confounding by injury timing and endocrine status; no external validation	Direct DoC-related prognostic candidate; ACTH may support 6-month consciousness recovery prediction	Wu et al. (2024)
11	Blood metabolomic signatures	Patients with brain injury-related DoC and healthy controls; *n* = 35 patients and 40 healthy controls	Case–control study	B, 3b	External validation absent	Small sample size; untargeted metabolomics; risk of overfitting; no independent validation cohort; limited prognostic outcome validation	Direct serum metabolomics discovery reference; 53 differential metabolites and 10 related pathways may support future biomarker screening and mechanism exploration	Feng et al. (2024)
12	CSF/serum/urine metabolomic panel	DoC patients classified as UWS or MCS; *n* = 51 total, including 35 UWS and 16 MCS patients; CSF/serum samples *n* = 49, urine samples *n* = 38	Cross-sectional study	B, 3b	External validation absent	Small sample size; unequal UWS/MCS groups; incomplete three-fluid sampling; high-dimensional metabolomics; risk of overfitting; no multicenter external validation	Direct DoC stratification candidate; CSF, serum, and urine metabolomic panels may help distinguish UWS from MCS but require larger multicenter validation	Wang et al. (2025)
13	Plasma/serum metabolomic and lipidomic markers	Chronic DoC patients after severe TBI and healthy controls; targeted metabolomics: *n* = 8 healthy controls, 12 VS, and 11 MCS; lipidomics: *n* = 32 healthy controls, 32 VS, and 22 MCS	Case–control study	B, 3b	External validation absent	Small subgroup sample size; TBI-only DoC cohort; single-center design; high-dimensional metabolomics/lipidomics; limited independent validation; potential overfitting in biomarker panel	Direct plasma/serum metabolomic and lipidomic reference; PC(38:5)-H and arachidonic acid may help distinguish VS from MCS, but require larger external validation	Yu et al. (2021)
14	Serum biochemical marker: TBIL	Patients with pDoC secondary to acquired brain injury; *n* = 139 included in final analysis	Retrospective cohort study	B, 2b	External validation absent	Single-center design; relatively small sample size; mixed etiologies; TBIL measured only once at baseline; no multiple-comparison correction; limited causal inference	Direct DoC prognostic candidate; higher serum TBIL was associated with better recovery at 1, 3, and 6 months, but requires prospective external validation	Huang et al. (2024)
15	Serum metabolomic signatures	Patients with pDoC secondary to acquired brain injury; *n* = 139 included in final analysis	Retrospective cohort study	B, 2b	External validation absent	Single-center design; relatively small sample size; mixed etiologies; TBIL measured only once at baseline; no multiple-comparison correction; limited causal inference	Direct DoC prognostic candidate; higher serum TBIL was associated with better recovery at 1, 3, and 6 months, but requires prospective external validation	Ge et al. (2024)
16	Serum miRNAs: miR-93, miR-191, miR-499	TBI patients and healthy controls; *n* = 76 TBI patients and 38 controls; TBI severity: mild *n* = 25, moderate *n* = 26, severe *n* = 25	Case–control study	B, 3b	Not validated in DoC	Not DoC-specific; relatively small sample size; TBI-only cohort; no multivariate adjustment; short miRNA monitoring period; possible extracerebral miRNA sources	Indirect TBI miRNA reference; serum miR-93, miR-191, and miR-499 may support TBI diagnosis, severity assessment, and prognosis, but remain indirect for DoC	Yang et al. (2016)
17	Blood miRNAs: miR-425-5p, miR-502, miR-21, miR-335	Patients with mTBI or sTBI plus extracranial injury, extracranial-injury controls, and healthy volunteers; discovery cohort *n* = 15; validation cohort *n* = 120, with 30 participants in each validation subgroup	Prospective cohort study	B, 2b	Not validated in DoC	Not DoC-specific; TBI-only cohort; small discovery set; GCS-based severity classification; extracranial injury confounding; risk of overfitting in prognostic modeling	Indirect TBI miRNA reference; miR-425-5p and miR-502 may support early mTBI diagnosis, while miR-21 and miR-335 may support sTBI assessment	Di Pietro et al. (2017)
18	Plasma miRNAs: miR-142-3p, miR-423-3p	sTBI patients with or without TBI and healthy volunteers; *n* = 38 total, including 33 trauma patients and 5 healthy volunteers	Retrospective cohort study	B, 3b	Not validated in DoC	Not DoC-specific; small sample size; trauma/TBI cohort only; isolated TBI group older than comparison groups; possible confounding by polytrauma, alcohol, sedation, shock, and blood-product administration	Indirect TBI miRNA reference; serum miR-423-3p may help identify severe isolated TBI early after trauma	Schindler et al. (2020)
19	Serum miRNA panel: seven miRNAs, especially miR-219a-5p	Patients with Mtbi/sTBI and healthy controls; screening cohort *n* = 30; validation cohort *n* = 244	Case–control study	B, 3b	Not validated in DoC	Not DoC-specific; TBI-only cohort; single-center design; no randomization or blinding; no predetermined sample-size calculation; serum miRNA normalization may affect reproducibility	Indirect TBI miRNA reference; seven serum miRNAs may support TBI diagnosis, while miR-219a-5p may help distinguish mild from sTBI and relate to prognosis	Yan et al. (2019)
20	DoC-related circulating miRNAs: miR-150-5p, miR-132-3p, miR-23b-3p, miR-451a, miR-16-5p	Patients with pDoC and healthy controls; patient cohort *n* = 30	Longitudinal multicenter cohort study	B, 2b	External validation absent	Small sample size; traumatic and hypoxic–ischemic etiologies; limited biomarker panel; outcome associations mainly exploratory	Direct DoC miRNA reference; miR-132-3p and miR-23b-3p may support prognostic assessment, especially in traumatic etiology	Musso et al. (2023)
21	Plasma/CSF miRNAs: miR-21-5p, miR-93-5p, miR-191-5p, let-7b-5p, miR-499-5p	Patients with pDoC and controls; *n* = 46 patients and 10 controls	Case–control study	B, 3b	External validation absent	Small sample size; mixed etiologies; cerebrospinal fluid and plasma findings not sufficient to distinguish VS/UWS from MCS; limited prognostic validation	Direct DoC miRNA reference; miR-93, miR-21, and miR-191 may reflect etiology-related differences rather than consciousness-state classification	Petrova et al. (2022)
22	Whole-blood miRNA profile	Patients with DoC after sTBI and controls; *n* = 6 patients and 5 controls	Case–control study	C, 4	External validation absent	Very small sample size; sTBI only; chronic-stage cohort; exploratory miRNA sequencing; limited generalizability	Direct DoC-TBI miRNA discovery reference; whole-blood miRNA profiles may help define neurobehavioral phenotypes	Zilliox et al. (2023)
23	Serum exosomal miRNAs: miR-206, miR-549a-3p	Patients with mTBI or sTBI and controls; *n* = 45 total	Case–control study	C, 4	External validation absent	Not DoC-specific; small sample size; TBI only; exosome isolation and normalization may affect reproducibility; limited outcome validation	Indirect TBI exosomal miRNA reference; serum exosomal miR-206 and miR-549a-3p may support TBI biomarker screening	Yang et al. (2024)
24	CSF protein biomarkers: CSF-TP, NSE	Patients with DoC after craniocerebral injury; *n* = 200	Cohort study	B, 2b	External validation absent	Single-center design; craniocerebral injury only; cerebrospinal fluid protein affected by hydrocephalus and treatment; limited molecular specificity	Direct DoC cerebrospinal-fluid protein reference; higher total cerebrospinal fluid protein may be associated with worse consciousness state and hydrocephalus risk	Liu et al. (2024)
25	CSF metabolomic signatures	Patients with DoC; *n* = 51	Cross-sectional study	B, 2b	External validation absent	Single-center design; mixed etiologies; cerebrospinal fluid metabolomics only; small subgroup size; limited external validation	Direct DoC cerebrospinal-fluid metabolomics reference; altered acylcarnitines, glutamate/GABA-related metabolites, and phospholipids may support etiological, diagnostic, and prognostic stratification	Xu et al. (2023)
26	Blood/CSF biomarker categories: neuronal, glial, inflammatory, metabolic	Blood and cerebrospinal fluid biomarker evidence in DoC	Narrative review	D, 5	Not applicable	Not an original cohort study; evidence synthesized from heterogeneous acute brain injury and DoC studies; no formal biomarker validation	Background reference; summarizes neuronal, glial, inflammatory, and metabolic biomarker categories for DoC research	Migdady et al. (2025)
27	Fecal microbiota and SCFAs	Patients with pDoC after sTBI; discovery cohort *n* = 43, validation cohort *n* = 44	Case–control study	B, 3b	Internally validated DoC candidate	TBI-only cohort; fecal microbiome affected by antibiotics, nutrition, infection, and gastrointestinal factors; causal inference limited	Direct DoC gut microbiome reference; Faecalibacterium, Enterococcus, and Methanobrevibacter may help distinguish MCS from VS	Yu et al. (2022)
28	CSF/serum/urine metabolomic markers	Patients with traumatic and non-traumatic DoC; *n* = 53, including 24 traumatic DoC and 29 non-traumatic DoC patients	Cross-sectional study	C, 4	External validation absent	Small sample size; mixed non-traumatic etiologies; high-dimensional metabolomics; very high AUC values require caution; no independent validation	Direct multi-fluid metabolomics reference; cerebrospinal fluid, serum, and urine metabolic panels may help differentiate traumatic from non-traumatic DoC	Xiao et al. (2024)
29	Salivary miRNAs	Human TBI studies using salivary miRNAs; nine included studies	Systematic review	B, 3a	Not validated in DoC	TBI-focused evidence; heterogeneous populations, saliva collection methods, and microRNA detection platforms; meta-analysis not feasible	Indirect salivary microRNA reference; saliva-based miRNAs may support future non-invasive biomarker research but remain exploratory for DoC	Hiskens et al. (2022)
30	Oral microbiome signatures	Patients with pDoC; *n* = 89	Case–control study	B, 3b	External validation absent	Single-center design; oral microbiome affected by antibiotics, oral care, enteral nutrition, and infection; causal inference limited	Direct DoC oral microbiome reference; oral microbial panels may help distinguish consciousness states and support prognostic modeling	Xu et al. (2025)

Evidence levels were assigned with reference to the Oxford center for Evidence-Based Medicine Levels of Evidence framework (March 2009; Oxford Centre for Evidence-Based Medicine, 2009). Classification was based primarily on study design, and was further interpreted in relation to directness to DoC populations, validation status, methodological limitations, and translational plausibility. Validation status was determined according to whether the biomarker evidence was directly tested in DoC populations and whether internal or external validation was available. “Not validated in DoC” indicates evidence derived from non-DoC or related brain-injury populations without direct validation in DoC patients; “External validation absent” indicates preliminary DoC-related evidence without confirmation in an independent cohort or multicenter population; “Internally validated DoC candidate” indicates a DoC-related candidate supported by validation within the same study or cohort but lacking independent external validation; “Preclinical only” indicates animal or laboratory evidence without human DoC validation; and “Not applicable” was used for reviews or background methodological papers without original biomarker validation.

### 6.2 Recurrent biological processes in DoC biomarker studies

Across the reviewed studies, biomarker findings can be organized into several recurrent mechanistic domains: structural neural injury, glial activation and blood–brain barrier dysfunction, neuroinflammatory responses, metabolic and bioenergetic disturbances, post-injury molecular regulation, and gut–brain axis-related systemic modulation.

These domains are clinically relevant because recovery of consciousness depends on the integrity and reactivation of distributed neural systems, including ascending arousal pathways, central thalamic systems, thalamocortical projections, frontoparietal associative networks, and cortico–striatal loops (Schiff, 2010; Snider et al., 2020; Mashour et al., 2020; Edlow et al., 2021). Severe brain injury may disrupt these systems through long-range axonal disconnection, impaired excitatory thalamocortical output, synaptic failure, astroglial and blood–brain barrier injury, secondary neuroinflammation, energy failure, neurotransmitter imbalance, and altered molecular regulation (Hill et al., 2016; Ng and Lee, 2019; Freire et al., 2023). Biomarkers become meaningful for DoC when they can be placed within these biological pathways and linked to the functional status of consciousness-supporting networks, rather than interpreted only as nonspecific molecular changes after brain injury.

Because these recurrent processes include both general consequences of severe brain injury and mechanisms more directly related to consciousness-supporting networks, they provide a basis for distinguishing markers that mainly reflect injury burden from those that may be closer to DoC-related pathophysiological processes. GFAP, UCH-L1, NSE, S100B, CSF total protein, and inflammatory mediators are biologically meaningful because they reflect astroglial injury, neuronal damage, blood–brain barrier disruption, or secondary neuroinflammatory cascades (Papa et al., 2016; Thelin et al., 2017; Sulhan et al., 2020; Mondello et al., 2012; Cash and Theus, 2020). However, these signals are not specific to impaired consciousness and may be influenced by lesion burden, extracranial injury, hemorrhage, infection, systemic inflammatory processes, sampling time, and the evolving post-injury clinical context (Thelin et al., 2017; Janigro et al., 2022). Their overlap with TBI and other forms of severe brain injury is not necessarily a weakness, because these conditions share pathogenic mechanisms and often lead to DoC. The limitation is that such biomarkers may primarily reflect the biological consequences of injury rather than the mechanisms underlying persistent impaired consciousness.

In contrast, biomarkers linked to persistent axonal disconnection, synaptic dysfunction, metabolic suppression, and recovery-related molecular regulation may have greater relevance to DoC-specific stratification, although none should currently be regarded as disease-specific without appropriate comparator cohorts. Their DoC-specific value will depend on whether they can distinguish patients with persistent impaired consciousness from brain-injured patients with comparable injury severity but preserved or recovered consciousness. For this reason, brain-injured patients without persistent DoC are more informative comparator groups than healthy controls when testing whether a biomarker is associated with impaired consciousness rather than trauma-related injury or brain damage itself. Thus, greater DoC relevance should be attributed not to markers that merely increase after TBI or severe brain damage, but to markers that remain associated with consciousness state, residual responsiveness, or recovery trajectory after controlling for injury severity, etiology, sampling time, and systemic complications.

### 6.3 Biomarkers related to neural connectivity, metabolism, and recovery

Among the reviewed candidates, biomarkers reflecting axonal integrity, synaptic function, metabolic competence, and post-injury molecular regulation may be more informative for DoC stratification than markers that primarily indicate nonspecific tissue injury or systemic responses.

Serum NfL is particularly relevant to pDoC because persistent axonal degeneration may indicate continued disruption of long-range connectivity required for network integration, although it still reflects structural injury burden and cannot alone define consciousness phenotype or recovery trajectory (Bagnato et al., 2021). CSF synaptic protein signatures, such as the YWHAG/NPTX2 ratio, may provide a more CNS-proximal signal related to synaptic dysfunction and outcome-associated neural changes (Oh et al., 2025), because consciousness recovery requires not only preserved anatomical pathways but also functional reactivation and communication within thalamocortical and associative networks (Lu et al., 2025; Cacciatore et al., 2025); however, this candidate still requires direct validation in DoC cohorts.

Metabolomic signatures may further complement structural and synaptic markers. They may reflect the biochemical conditions that support consciousness-related network activity, including energy availability, neurotransmitter balance, membrane integrity, oxidative stress regulation, and astrocyte–neuron metabolic coupling (Xu et al., 2023; Yu et al., 2021; Chikahisa and Séi, 2011; Bélanger et al., 2011). Coordinated miRNA patterns may add a regulatory layer if they reflect dynamic post-injury molecular regulation associated with secondary injury and recovery-related transitions (Musso et al., 2023; Petrova et al., 2022; Zilliox et al., 2023).

By contrast, fecal SCFAs, microbiota-derived metabolites, urinary metabolites, and salivary signatures remain more indirect candidates because their relationship with consciousness-supporting networks is mediated through systemic pathways and is strongly influenced by diet, antibiotics, enteral nutrition, infection, gastrointestinal function, renal handling, hydration, oral status, medication exposure, and systemic illness (Yu et al., 2022; Silva et al., 2020; Kurhaluk et al., 2025; Seol et al., 2020; Stevens et al., 2019).

Overall, serum NfL, CSF synaptic protein signatures, selected metabolomic profiles, and coordinated miRNA patterns appear to be the most promising candidate categories at this stage, because they are more closely related to neural connectivity, synaptic communication, metabolic competence, and post-injury regulatory processes than markers that mainly reflect systemic or specimen-specific changes.

### 6.4 Stratified biomarker evaluation and longitudinal monitoring

These mechanistic distinctions suggest that DoC biomarker research should shift from isolated candidate markers toward purpose-defined biomarker panels stratified by etiology, disease stage, and consciousness state.

In acute or subacute TBI-related DoC, an axonal–glial–inflammatory panel including NfL, GFAP, UCH-L1, S100B, inflammatory mediators, and selected miRNAs could be tested for its ability to distinguish persistent DoC from severe TBI without prolonged consciousness impairment and to predict transition from UWS to MCS. In HIBI-related DoC, panels emphasizing metabolic and inflammatory vulnerability may be more appropriate because hypoxic injury can impair energy-dependent synaptic activity, produce diffuse neuronal stress, and reduce the capacity for network reactivation (Busl and Greer, 2010); such panels may include NfL, inflammatory mediators, and serum or CSF metabolites related to amino acid neurotransmission, purine or adenosine metabolism, phospholipid remodeling, and oxidative stress.

In chronic UWS and MCS, longitudinal combinations of NfL, CSF synaptic protein signatures, selected miRNAs, and metabolite panels should be evaluated for their associations with residual network integrity, consciousness-state transitions, delayed recovery, and long-term functional outcome (Edlow et al., 2021; Musso et al., 2023; Bagnato et al., 2021; Xu et al., 2023). In MCS+ or recovery-stage patients, biomarker panels may be most useful as adjunctive tools when behavioral responses are inconsistent or difficult to interpret, rather than as replacements for established behavioral, electrophysiological, or neuroimaging.

Future evaluation of these panels should be conducted in prospectively defined cohorts with standardized sampling windows, clearly specified etiologies, predefined consciousness states, and clinically meaningful endpoints, including UWS/MCS differentiation, emergence from MCS, delayed recovery, long-term functional outcome, and incremental value beyond established behavioral, electrophysiological, neuroimaging, and clinical prognostic assessments. Whenever possible, comparator groups should include brain-injured patients without persistent DoC, allowing biomarker associations with impaired consciousness to be distinguished from associations with injury severity alone.

### 6.5 Evidence limitations and clinical implementation requirements

The current evidence remains limited by small sample sizes, single-center designs, methodological heterogeneity, inconsistent sampling windows, and insufficient external validation. Differences in sample preprocessing, assay platforms, normalization strategies, metabolite identification, miRNA quantification, and statistical modeling limit cross-study comparability, while many metabolomic, miRNA, fecal, urinary, and salivary models with high reported performance may be vulnerable to overfitting. Disease stage and etiology should be considered not only as stratification variables, but also as biological factors that influence biomarker expression, mechanistic interpretation, and prognostic value, because acute, subacute, prolonged, and chronic DoC may involve different combinations of primary tissue injury, secondary inflammation, blood–brain barrier dysfunction, systemic metabolic responses, synaptic remodeling, and long-term repair or neurodegenerative processes (Edlow et al., 2021; Bodien et al., 2026). Similarly, TBI-related DoC, HIBI-related DoC, vascular etiologies, and mixed injuries may differ in axonal injury, diffuse hypoxic neuronal damage, vascular injury, neuroinflammatory response, and network disruption patterns (Giacino et al., 2018; Edlow et al., 2021; Bodien et al., 2026).

Future studies should therefore move from exploratory associations toward coordinated validation and implementation-oriented research. Large-scale prospective multicenter cohorts are needed to evaluate the reproducibility, stability, and generalizability of candidate biomarkers across etiologies, consciousness states, disease stages, and patient populations. Such studies should include brain-injured patients without persistent DoC as comparator groups, harmonize biospecimen collection and preprocessing protocols, standardize assay platforms and outcome measures, and evaluate the incremental predictive value of candidate biomarkers or panels beyond established behavioral, electrophysiological, and neuroimaging assessments, while accounting for etiology, disease duration, and established prognostic factors. In early translational phases, clinically feasible approaches, such as ultrasensitive blood-based protein assays, may be more suitable for repeated monitoring and prognostic stratification than highly complex and costly multi-omics platforms. At the same time, mechanistic studies should further clarify the central–peripheral–intestinal interactions linking biomarker changes with consciousness-related neural circuits, neuroinflammation, metabolic disturbance, synaptic function, and recovery-related regulation.

**Figure 9:**
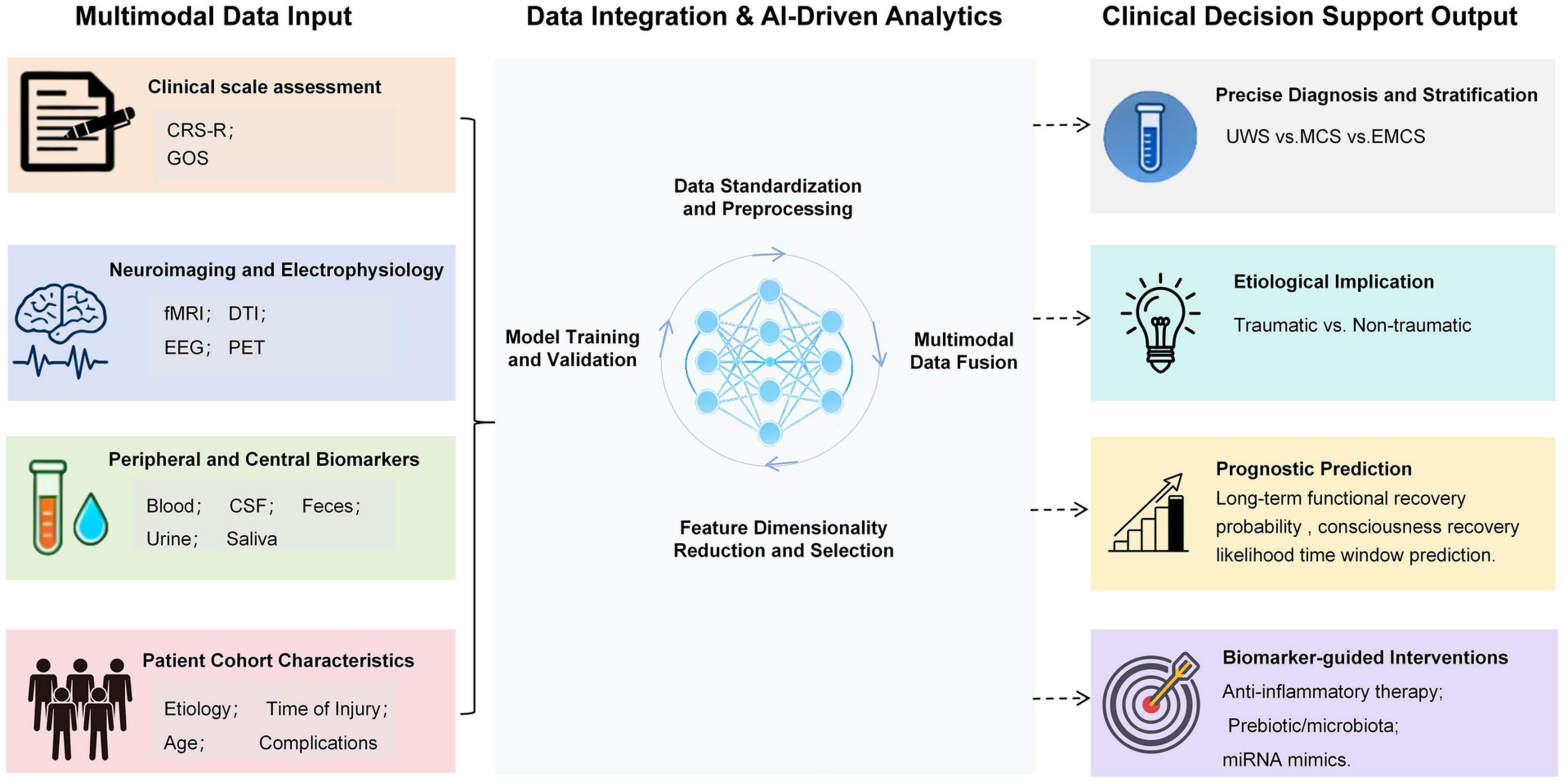
Multimodal data integration framework for DoC biomarker translation. This schematic illustrates a multimodal data integration framework for disorders of consciousness (DoC) assessment. The input layer includes clinical scale assessment, neuroimaging and electrophysiology, peripheral and central biomarkers, and patient cohort characteristics. The middle layer shows data standardization and preprocessing, feature dimensionality reduction and selection, model training and validation, and multimodal data fusion. The output layer includes clinical decision-support categories for precise diagnosis and stratification, etiological implication, prognostic prediction, and biomarker-guided interventions.

Multimodal and AI-assisted models may facilitate the integration of biomarker profiles with behavioral assessments, electrophysiology, neuroimaging, etiology, disease duration, and treatment response, but their clinical application requires multicenter datasets, external validation, model interpretability, external generalizability, and predefined clinical thresholds (Liuzzi et al., 2022; Bonanno et al., 2025; Shu et al., 2025; May et al., 2025; Orenuga et al., 2025). Figure 9 illustrates a proposed clinical decision-support framework in which clinical scale assessments, neuroimaging and electrophysiology, peripheral and central biomarkers, and cohort-level characteristics are first standardized and preprocessed, followed by model training, validation, feature selection, and multimodal data fusion. The resulting outputs may support precise diagnosis and stratification, etiological interpretation, prognostic prediction, and biomarker-guided intervention design. In practical implementation, predictive depth should be balanced with clinical feasibility: minimally invasive or non-invasive specimens, particularly blood and urine, may be prioritized for screening and longitudinal monitoring, whereas CSF and fecal biomarkers may be more appropriate for central validation, mechanism exploration, differential diagnosis, and targeted treatment development. Biomarker-informed interventions, such as microbiota modulation or miRNA-targeted strategies, remain exploratory and require rigorous preclinical validation and well-designed clinical trials before clinical application. Because large-scale biomarker research is resource-intensive, responsible translation will also require coordinated funding mechanisms, multicenter clinical networks, standardized data governance, shared biobanks, and prospective evaluation of clinical utility and cost-effectiveness. These steps are necessary before DoC biomarkers can be incorporated as reliable adjunctive tools into routine care.

## 7 Conclusion

This review highlights the value of interpreting DoC biomarkers within a central–peripheral–intestinal framework. Within this framework, blood-based markers may support dynamic monitoring and prognostic stratification, CSF-related markers may help define CNS-proximal pathological processes, and gut-related, urinary, and salivary markers remain exploratory indicators of systemic regulation. The strongest translational potential lies in multimodal panels that combine markers of axonal integrity, synaptic function, metabolic competence, and post-injury molecular regulation with established behavioral, electrophysiological, and neuroimaging assessments, rather than in any single universal biomarker. However, current evidence remains limited by insufficient validation across etiologies, disease stages, consciousness states, sampling windows, and brain-injured comparator cohorts. Future studies should prioritize prospective multicenter validation, standardized biospecimen protocols, longitudinal sampling, clinically interpretable thresholds, and clinically meaningful endpoints. Clinical translation will require evidence that DoC biomarkers are reproducible across cohorts and provide incremental information beyond established behavioral, electrophysiological, and neuroimaging assessments.

## Glossary

ACTH: Adrenocorticotropic hormone
AUC: Area under the curve
BDNF: Brain-derived neurotrophic factor
CSF: Cerebrospinal fluid
CSF-TP: Cerebrospinal fluid total protein
CMD: Cognitive motor dissociation
CNS: Central nervous system
CRS-R: Coma Recovery Scale-Revised
dPCR: Digital polymerase chain reaction
DoC: Disorders of consciousness
EMCS: Emerged from the minimally conscious state
ELISA: Enzyme-linked immunosorbent assay
fMRI: Functional magnetic resonance imaging
FMT: Fecal microbiota transplantation
GFAP: Glial fibrillary acidic protein
GBA: Gut-brain axis
GC × GC-TOFMS: Comprehensive two-dimensional gas chromatography–time-of-flight mass spectrometry
GOS-E: Glasgow Outcome Scale-Extended
HIBI: Hypoxic–ischemic brain injury
HPA: Hypothalamic–pituitary–adrenal
IL-6: Interleukin-6
LBP: Lipopolysaccharide-binding protein
LC–MS: Liquid chromatography-mass spectrometry
LC–MS/MS: Liquid chromatography–tandem mass spectrometry
LysoPC: Lysophosphatidylcholine
LysoPE: Lysophosphatidylethanolamine
LysoSM: Lysosphingomyelin
MCS: Minimally conscious state
miRNA/miRs: microRNA/microRNAs
mTBI: Mild traumatic brain injury
NF-H: Neurofilament heavy chain
NF-κB: Nuclear factor-κB
NfL: Neurofilament light chain
NSE: Neuron-specific enolase
NPTX2: Neuronal pentraxin 2
PI3K/AKT: Phosphatidylinositol 3-kinase/protein kinase B
pDoC: Prolonged disorders of consciousness
qPCR: Quantitative polymerase chain reaction
qRT-PCR: Quantitative reverse transcription-polymerase chain reaction
RANTES: Regulated on activation, normal T cell expressed and secreted
RT-PCR: Reverse transcription-polymerase chain reaction
RNA-seq: RNA sequencing
SCFAs: Short-chain fatty acids
S100B: S100 Calcium binding protein beta
Simoa: Single-molecule array
sTBI: Severe traumatic brain injury
TLR4: Toll-like receptor 4
TBI: Traumatic brain injury
TNF-α: Tumor necrosis factor-α
TLDA: TaqMan low-density arrays
16S rRNA: 16S ribosomal RNA
UCH-L1: Ubiquitin C-terminal hydrolase-L1
UPLC-MS/MS: Ultra-performance liquid chromatography–tandem mass spectrometry
UHPLC-Q-Orbitrap HRMS: Ultra-high-performance liquid chromatography coupled with quadrupole-Orbitrap high-resolution mass spectrometry
VS/UWS: Vegetative state/unresponsive wakefulness syndrome
YWHAG: Tyrosine 3-monooxygenase/tryptophan 5-monooxygenase activation protein gamma
