# Supplementary material for: Advances in biomarkers for diagnosing and prognosticating disorders of consciousness
Source: Front Neurosci. 2026 Jul 16;20:1823376. doi: 10.3389/fnins.2026.1823376 (PMC13422503; doi:10.3389/fnins.2026.1823376)
Supplement: Supplementary file 1 [file Table_1.DOCX]

**Supplementary Table S1. Search terms and representative search combinations used for the narrative review**

| **Search concept** | **Search terms** |
| --- | --- |
| Disorders of consciousness | “disorders of consciousness” OR “DoC” OR “prolonged disorders of consciousness” OR “pDoC” OR “vegetative state” OR “VS” OR “unresponsive wakefulness syndrome” OR “UWS” OR “minimally conscious state” OR “MCS” OR “MCS+” OR “MCS−” OR “emergence from minimally conscious state” OR “EMCS” |
| Brain injury | “traumatic brain injury” OR “TBI” OR “severe traumatic brain injury” OR “hypoxic-ischemic brain injury” OR “HIBI” OR “anoxic brain injury” OR “stroke” OR “cerebrovascular injury” OR “acquired brain injury” |
| Biomarkers | “biomarker” OR “biomarkers” OR “molecular marker” OR “diagnostic biomarker” OR “prognostic biomarker” OR “predictive biomarker” |
| Biological samples | “serum” OR “plasma” OR “blood” OR “peripheral blood” OR “cerebrospinal fluid” OR “CSF” OR “feces” OR “faeces” OR “stool” OR “urine” OR “saliva” OR “oral microbiome” |
| Molecular categories | “proteomics” OR “protein biomarker” OR “metabolomics” OR “metabolite” OR “microRNA” OR “miRNA” OR “exosomal miRNA” OR “cytokine” OR “inflammatory cytokine” OR “neurofilament light chain” OR “NfL” OR “GFAP” OR “UCH-L1” OR “S100B” |
| Gut-brain axis | “gut microbiota” OR “gut microbiome” OR “gut-brain axis” OR “short-chain fatty acids” OR “SCFAs” OR “fecal microbiota transplantation” OR “FMT” |

**Representative search combinations：**

**Search combination 1: DoC and multi-sample biomarkers**
(“disorders of consciousness” OR “prolonged disorders of consciousness” OR “vegetative state” OR “unresponsive wakefulness syndrome” OR “minimally conscious state” OR “MCS” OR “DoC”) AND (“biomarker” OR “biomarkers” OR “proteomics” OR “metabolomics” OR “microRNA” OR “miRNA”) AND (“serum” OR “plasma” OR “blood” OR “cerebrospinal fluid” OR “CSF” OR “feces” OR “faeces” OR “stool” OR “urine” OR “saliva”)

**Search combination 2: Brain injury-related biomarker evidence**
(“traumatic brain injury” OR “severe traumatic brain injury” OR “hypoxic-ischemic brain injury” OR “anoxic brain injury” OR “acquired brain injury”) AND (“biomarker” OR “blood-based biomarker” OR “GFAP” OR “UCH-L1” OR “S100B” OR “NfL” OR “metabolomics” OR “microRNA”) AND (“diagnosis” OR “prognosis” OR “outcome” OR “recovery”)

**Search combination 3: Gut-brain axis and fecal biomarkers**
(“disorders of consciousness” OR “prolonged disorders of consciousness” OR “minimally conscious state” OR “unresponsive wakefulness syndrome”) AND (“gut microbiota” OR “gut microbiome” OR “gut-brain axis” OR “short-chain fatty acids” OR “SCFAs” OR “fecal biomarker” OR “fecal metabolomics”)

**Search combination 4: Non-invasive sample biomarkers**
(“disorders of consciousness” OR “brain injury” OR “traumatic brain injury” OR “acquired brain injury”) AND (“urine” OR “saliva” OR “oral microbiome” OR “non-invasive biomarker”) AND (“biomarker” OR “metabolomics” OR “microRNA” OR “protein biomarker”)
