# Supplementary figures and images for: Advances in biomarkers for diagnosing and prognosticating disorders of consciousness
Source: Front Neurosci. 2026 Jul 16;20:1823376. doi: 10.3389/fnins.2026.1823376 (PMC13422503; doi:10.3389/fnins.2026.1823376)

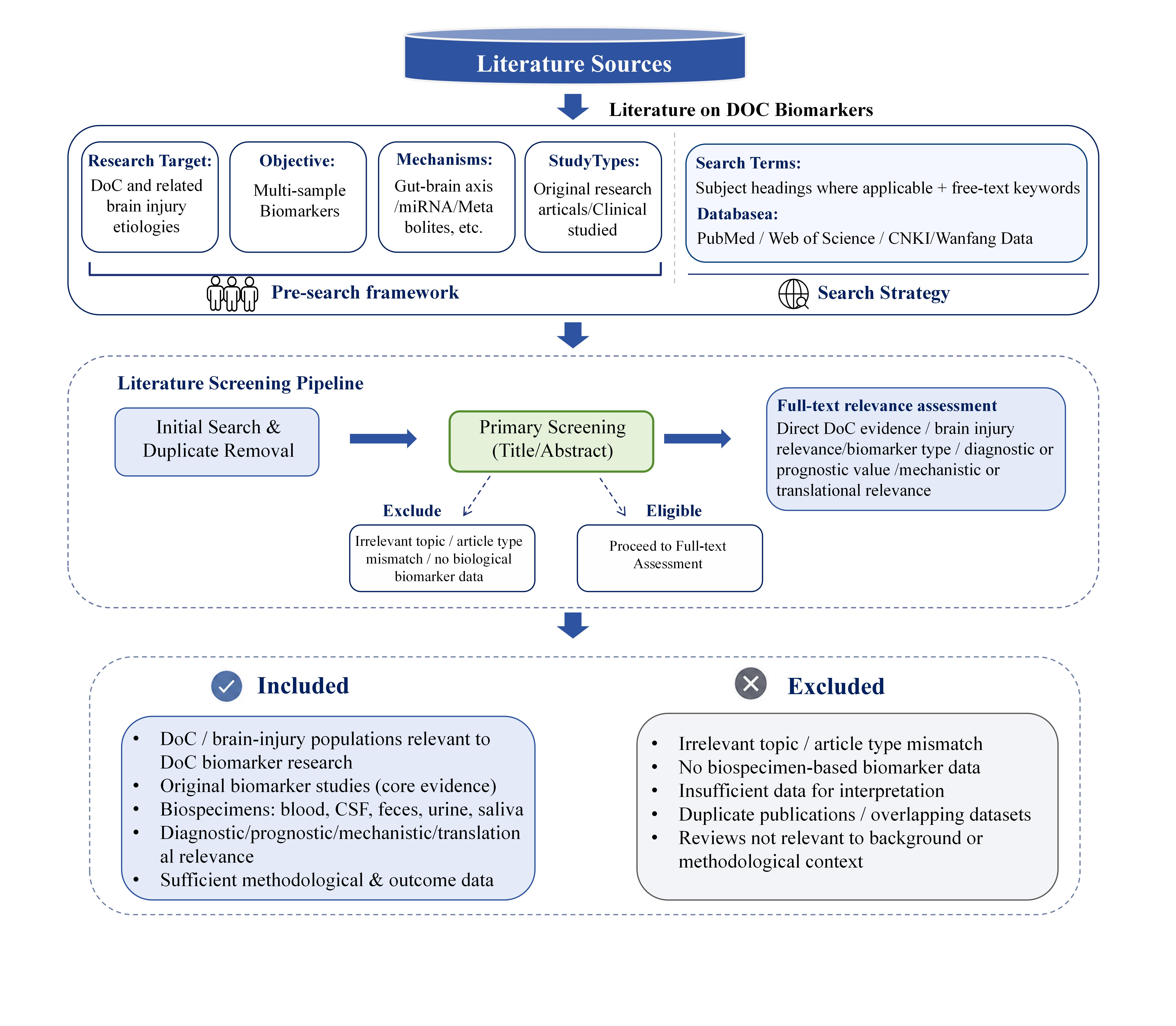

Supplement: Supplementary file 2 [file Image_1.JPEG]
